# Synthesis and properties of [8]-, [10]-, [12]-, and [16]cyclo-1,4-naphthylenes†
†Electronic supplementary information (ESI) available: Detailed experimental procedures, computational studies, and spectral data for all compounds. See DOI: 10.1039/c6sc04048a


**DOI:** 10.1039/c6sc04048a

**Published:** 2016-09-12

**Authors:** Keishu Okada, Akiko Yagi, Yasutomo Segawa, Kenichiro Itami

**Affiliations:** a Graduate School of Science , Nagoya University , Chikusa , Nagoya 464-8602 , Japan . Email: ysegawa@nagoya-u.jp ; Email: itami@chem.nagoya-u.ac.jp; b JST , ERATO , Itami Molecular Nanocarbon Project , Nagoya University , Japan; c Institute of Transformative Bio-Molecules (WPI-ITbM) , Nagoya University , Japan

## Abstract

The synthesis and properties of various [*n*]cyclo-1,4-naphthylenes ([*n*]CNs, *n* = 8, 10, 12, and 16) are described. Initially, extended L-shaped units, which could be converted into quater- or quinquenaphthylenes were prepared. Nickel- or palladium-mediated couplings of these extended L-shaped units, followed by reductive aromatization of the coupling products afforded [8]-, [10]-, [12]-, and [16]CNs. The size-dependent photophysical properties of these CNs were confirmed by measuring their UV-vis absorption and fluorescence spectra. The theoretical studies supported substantial effects of the number of naphthalene rings on the structural and photophysical properties of these CNs. A kinetic study on the thermal conversion of the *C*_s_-symmetric conformer of [10]CN into its most stable *D*_5d_-symmetric conformer indicated that ring strain affects the rotation barrier of the naphthalene rings in [10]CN.

## Abstract 2

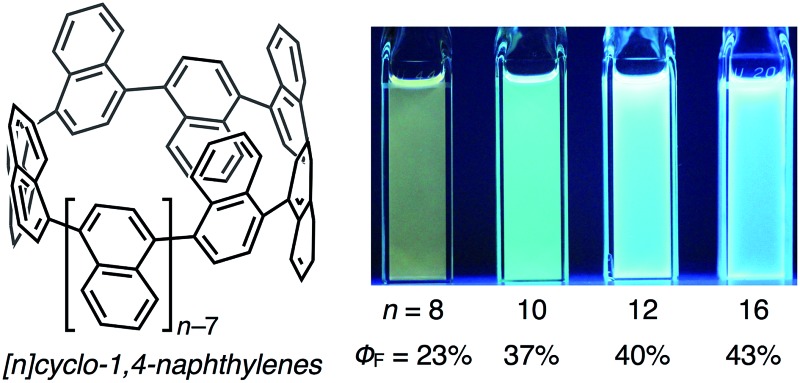
The synthesis and properties of various [*n*]cyclo-1,4-naphthylenes ([*n*]CNs, *n* = 8, 10, 12, and 16) are described.

## Introduction

**Fig. 1:**
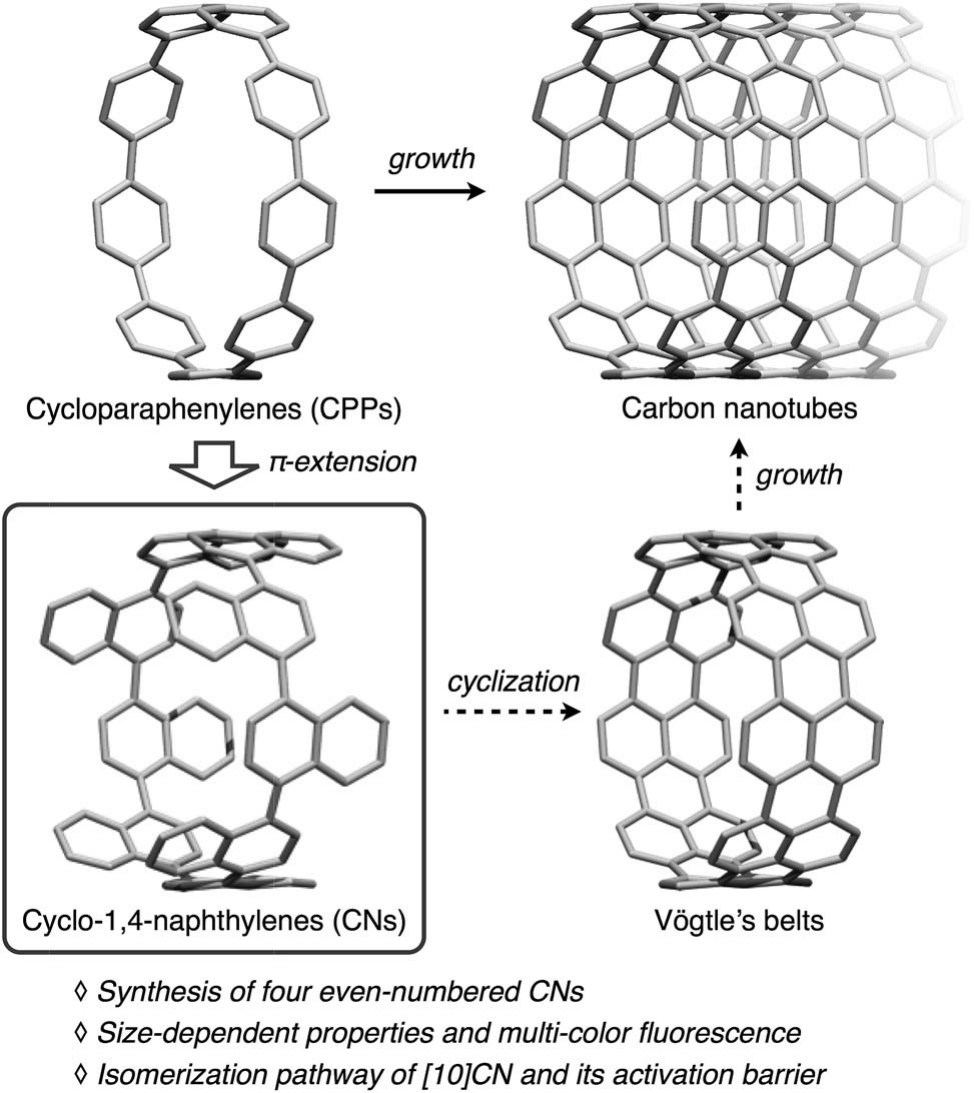
CPPs, CNs, and Vögtle's belts as the sidewall segments of CNTs.

Cycloparaphenylenes^1^ (CPPs, Fig. 1), *i.e.*, ring-shaped aromatic hydrocarbons consisting exclusively of paraphenylenes, have recently received much attention on account of their highly symmetric structures and the unique electronic properties that arise from their radial π-conjugation modes. CPPs have also been proposed as building blocks for carbon nanotubes (CNTs), given that CPPs represent the shortest sidewall segment of armchair CNTs.^2^ Since the first synthesis of [9]-, [12]-, and [18]CPPs by Bertozzi and Jasti in 2008,^3*a*^ CPPs of various size, *i.e.*, [5]–[18]CPPs have been synthesized by the Jasti,^3^ Itami,^4^ and Yamago^5^ research groups. While the amounts of synthesized CPPs were very small in the beginning, synthetic improvements allowed syntheses on the gram-scale,^3e,5g^ and several CPP sizes are now commercially available.^6^

With CPPs of various sizes in hand, unprecedented size-dependencies of the physical properties of CPPs have been revealed and size-specific applications of the CPPs have been discovered. For example, the HOMO–LUMO gap of the CPPs increases with increasing CPP sizes, which stands in contrast to the size-dependency of linear oligoparaphenylenes. As a consequence, the [*n*]CPPs show size-dependent fluorescence properties.^5b,7^ The selective incorporation of fullerenes into [*n*]CPPs (*n* = 10 and 11) was possible because the fullerenes fit into the cavity of these CPPs.^3e,8^ The uniform porous structure of [12]CPP renders it a discrete adsorbent of H_2_O, MeOH, and EtOH.^9*a*^ The diameter-controlled growth of CNTs has been achieved by using [9]- and [12]CPPs as seeds for chemical vapor deposition methods.^9*b*^ Strategies for the size-selective synthesis of CPPs have widely been applied to the synthesis of related ring-shaped^10^ or cage-shaped π-conjugated molecules.^11^ That is, the controlling the size of CPPs has allowed significant innovations in this field.

Among the derivatives of CPPs, cyclo-1,4-naphthylenes (CNs) are simple but intriguing molecules. CNs consist of naphthalene rings and can be considered as π-extended CPPs generated by benzannulation of all the benzene rings in CPPs. Our group previously reported the synthesis of [9]CN, and the unique structural properties of [9]CN attributed to the sterically hindered integration of naphthalene rings were uncovered.^12^ However, due to the lack of CNs of other sizes, the size-dependent properties and the odd–even effect in CNs have so far remained unexplored. Moreover, CNs are potential precursors for the so-called Vögtle's belts, a type of carbon nanobelts proposed by Vögtle in 1983.^13^ Since CNs contain all the carbon content required for Vögtle's belts, ideal cyclodehydrogenation reactions between the *peri* positions of neighboring naphthalene rings can convert CNs into the corresponding Vögtle's belts.^1m,14^ The rigid structures of Vögtle's have been proposed to be thermally stable seeds for the perfectly controlled growth of CNTs. The synthesis of CNs of varying size is thus an attractive research target in synthetic organic chemistry, as CNs are an interesting new family of radially π-conjugated molecules, and potentially also important for the tailored synthesis of CNTs.

Herein, we report the synthesis of even-numbered [*n*]CNs (*n* = 8, 10, 12, and 16) and their size-dependent photophysical properties, especially the multi-color fluorescence. The rotation barrier of the naphthalene rings in [10]CN was determined experimentally and theoretically.

## Results and discussion

### Synthesis of [8]-, [10]-, [12]-, and [16]CNs

**Fig. 2:**
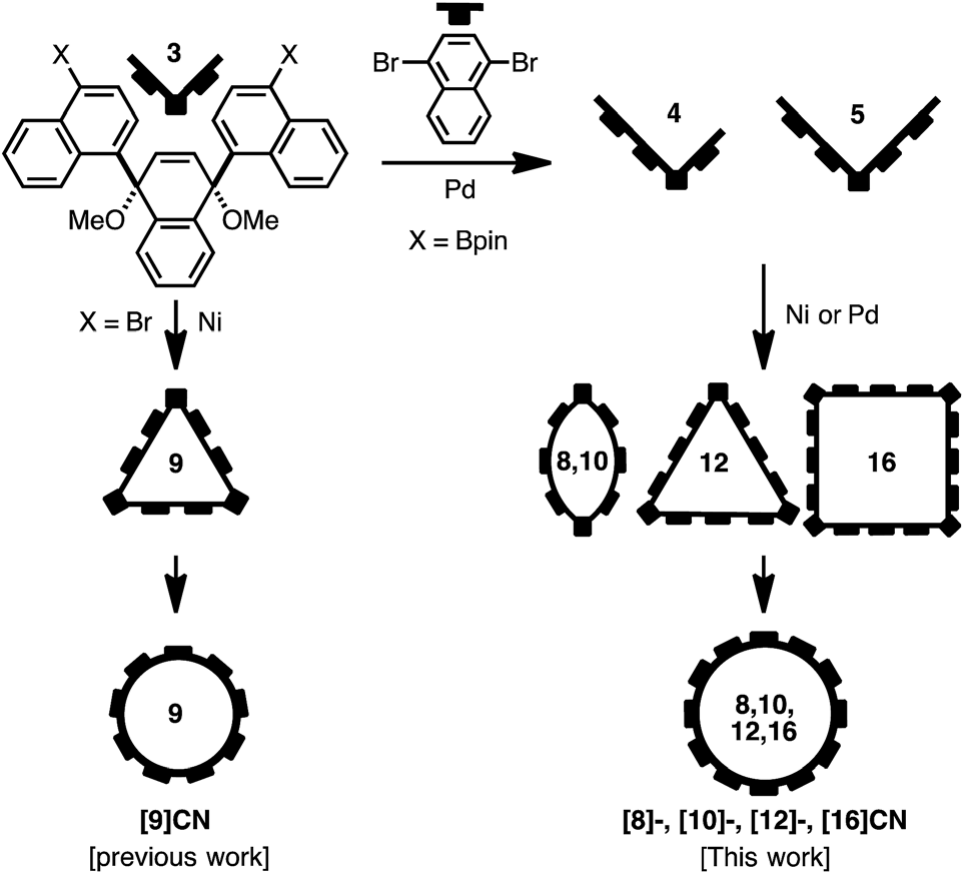
Synthetic route for CNs.

Our synthetic strategy for the generation of CNs is shown in Fig. 2. Previously we synthesized [9]CN through a nickel-mediated cyclotrimerization of the ternaphthyl-convertible L-shaped unit and a subsequent reductive aromatization.^12^ To obtain CNs of various sizes, we designed extended L-shaped units that can be converted into quater- or quinquenaphthylene units. A widely applied strategy in the synthesis of CPPs^3–5^ is the combination of L-shaped units with linear units that allows synthesizing macrocycles of varying size. A palladium-catalyzed Suzuki–Miyaura cross-coupling^15^ or a nickel-mediated homocoupling^16^ produced the desired macrocycles as precursor to CNs. In contrast to the synthesis of [9]CN, not only cyclic trimers but also cyclic dimers and tetramers were obtained.

**Scheme 1:**
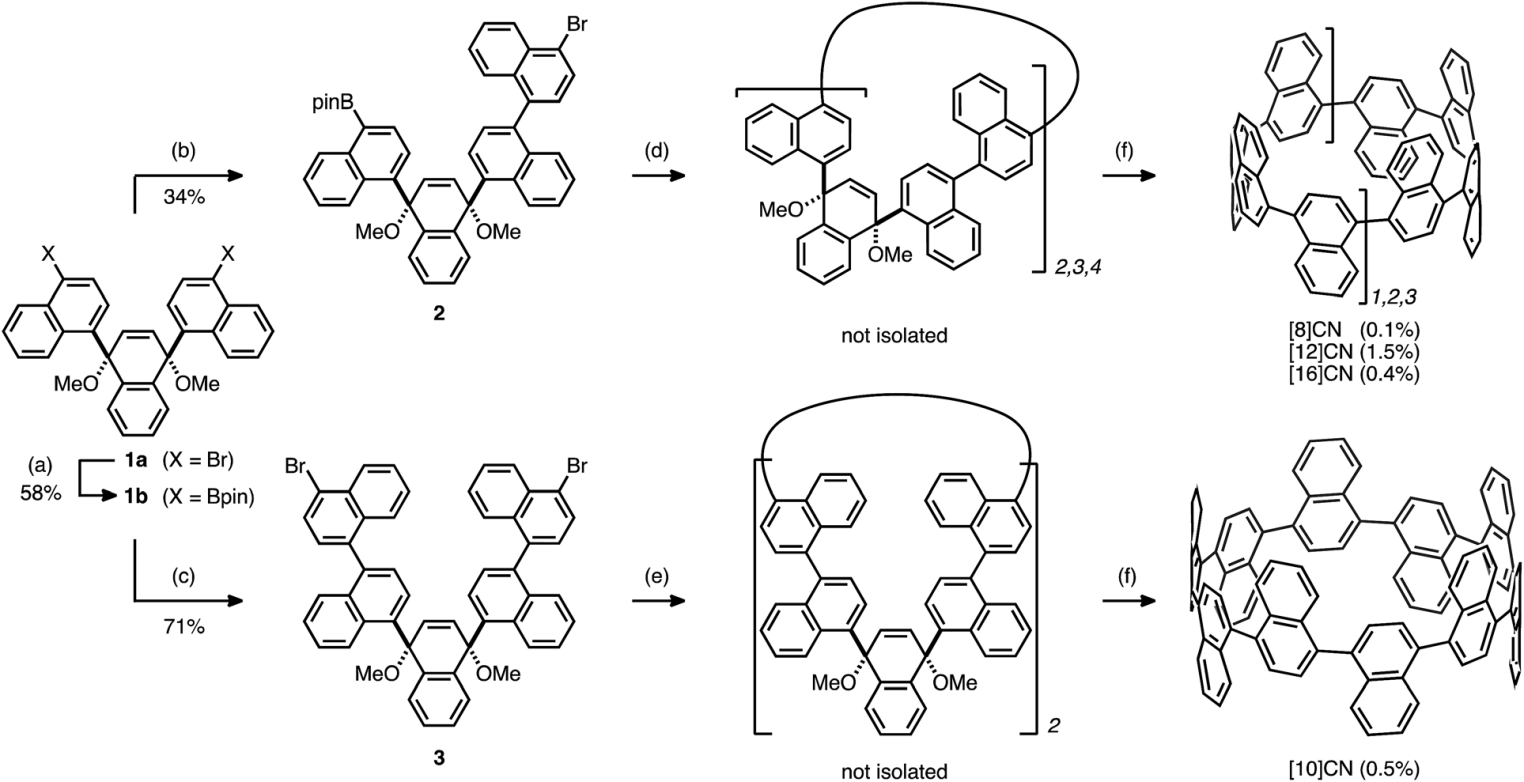
Synthesis of [8]-, [10]-, [12]-, and [16]CNs. Reaction conditions: (a) **1a** (1 equiv.), B_2_pin_2_ (2.5 equiv.), PdCl_2_(dppf)·CH_2_Cl_2_ (5 mol%), KOAc (6 equiv.), 1,4-dioxane, 90 °C, 10 h. (b) **1b** (1 equiv.), 1,4-dibromonaphthalene (2.5 equiv.), Pd(PPh_3_)_4_ (10 mol%), K_2_CO_3_ (5 equiv.), toluene/EtOH/H_2_O, 90 °C, 5 h. (c) **1b** (1 equiv.), 1,4-dibromonaphthalene (5 equiv.), Pd(PPh_3_)_4_ (10 mol%), K_2_CO_3_ (5 equiv.), toluene/EtOH/H_2_O, 90 °C, 16 h. (d) **2** (1 equiv.), Pd(PPh_3_)_4_ (10 mol%), K_2_CO_3_ (5 equiv.), toluene/EtOH/H_2_O, 90 °C, 26 h. (e) **3** (1 equiv.), Ni(cod)_2_ (2.2 equiv.), 2,2′-bipyridyl (2.2 equiv.), DMF, 90 °C, 24 h. (f) Li (excess), THF, rt, 12 h. Bpin = 4,4,5,5-tetramethyl-1,3,2-dioxaborolan-2-yl.

Initially, we prepared the extended L-shaped units by sequential coupling reactions (Scheme 1). Diboryl L-shaped unit **1b** was synthesized by a palladium-catalyzed Miyaura borylation^17^ of dibromo L-shaped unit **1a** in 58% yield. A subsequent Suzuki–Miyaura cross-coupling reaction between **1b** and 2.5 equiv. of 1,4-dibromonaphthalene furnished the quaternaphthylene-convertible unit **2** in 34% yield. Under the same reaction conditions, except for an excess of 1,4-dibromonaphthalene (5.0 equiv. with respect to **1b**), quinquenaphthylene-convertible unit **3** was obtained in 71% yield.

In order to generate cyclic oligomers, **2** was subjected to Suzuki–Miyaura cross-coupling reaction conditions (Scheme 1): **2** was dissolved in a mixed solvent system (toluene/EtOH/H_2_O; 25 mM) and heated to 90 °C for 26 hours in the presence of 10 mol% Pd(PPh_3_)_4_ and 5 equiv. of K_2_CO_3_. The products were purified by preparative recycling gel permeation chromatography (GPC) to afford a mixture of cyclic oligomers. Because further purification proved difficult, the mixture was subjected to the reductive aromatization using granular lithium. As a result, [8]-, [12]-, and [16]CN were successfully isolated in 0.1%, 1.5%, and 0.4% yields, respectively (yields with respect to **2**). This result indicates that the cross-coupling of **2** produced the corresponding cyclic dimer, trimer, and tetramer in low yields, whereas the homocoupling of **1a** only afforded the cyclic trimer.^12^

The other L-shaped unit, **3**, is also a precursor for the synthesis of CNs. Treatment of **3** (1.0 mM) with Ni(cod)_2_ (2.2 equiv.) and 2,2′-bipyridyl (2.2 equiv.) in DMF at 85 °C furnished a mixture of coupling products. After the following reductive aromatization, [10]CN was isolated in 0.5% yield over two steps.^18^ This result indicates that the mixture of coupling products contained a small amount of cyclic dimer.

**Fig. 3:**
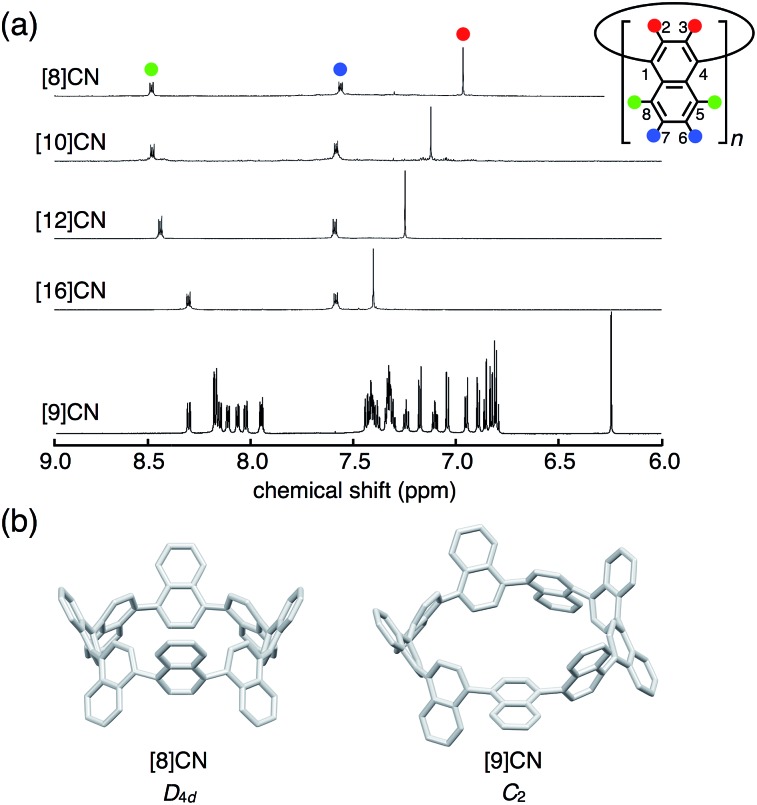
(a) The ^1^H NMR spectra of [*n*]CNs (*n* = 8, 9, 10, 12, 16) in CD_2_Cl_2_ with the corresponding assignment of signals. (b) Structures of [8]- and [9]CNs with their symmetry optimized at B3LYP/6-31G(d) level of theory.

The ^1^H NMR spectra of [8]-, [10]-, [12]-, and [16]CNs in CD_2_Cl_2_ displayed a clear size-dependency (Fig. 3a). These even-numbered CNs showed simple spectra with three types of proton signals assigned to the 2,3- (red), 5,8- (green), and 6,7-positions (blue) of the naphthalene units. These spectra indicated highly symmetric structures for [8]-, [10]-, [12]-, and [16]CNs in solution, which stands in contrast to that of [9]CN. Highly symmetric conformations (such as *D*_4d_ for [8]CN, Fig. 3b) were optimized by DFT calculations as the most stable ones, and these were in good agreement with the highly symmetric NMR spectra. The NMR signals for the hydrogen atoms at the 2,3-positions (red) of the naphthalene units shifted upfield with decreasing size of [*n*]CN. DFT calculations indicated that the size-dependent upfield shifts should mainly arise from the through-space shielding of neighboring naphthalene rings, which increases as the bent angles of naphthalene rings increases (for details, see ESI†). A similar trend was observed in [*n*]CPPs (*n* = 8–18).^1*k*^

### Photophysical properties of CNs

**Fig. 4:**
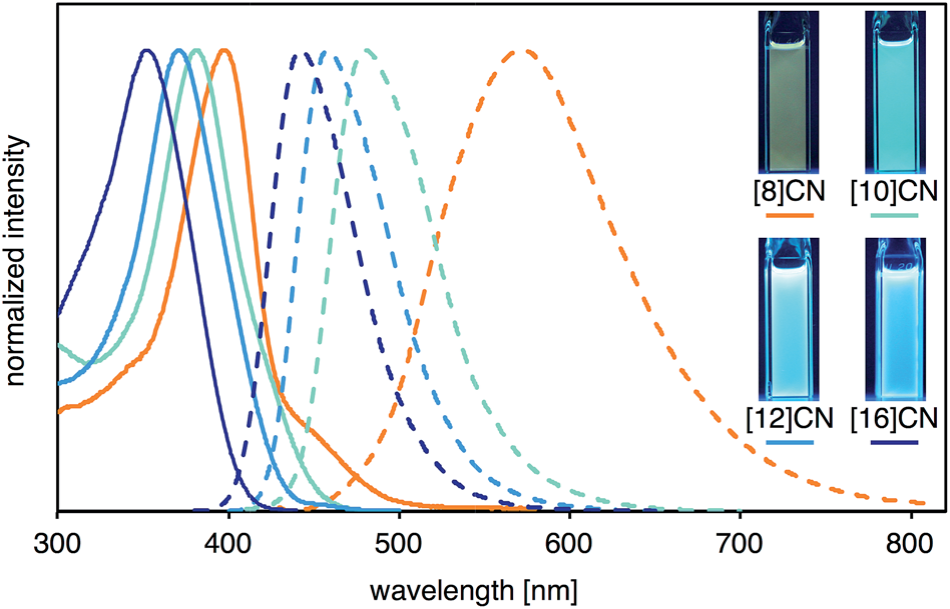
UV-vis absorption (solid line) and fluorescence (broken line) spectra of CNs with photographic images of the CH_2_Cl_2_ solutions under UV irradiation at *λ* = 365 nm.

The photophysical properties including UV-vis absorption and fluorescence of [8]-, [10]-, [12]-, and [16]CNs were investigated. The spectra of these CNs in CH_2_Cl_2_ are shown in Fig. 4, and the photophysical data are summarized in Table 1. In the UV-vis absorption spectra, the maximum-wavelength absorption bands (*λ*_abs1_) shifted to shorter wavelengths with increasing ring size. This behavior is clearly different from [*n*]CPPs (*n* = 6–18), which have absorption maxima between 338–340 nm independently of their size.^1j,7^ The peak wavelengths of the shoulder-shaped absorption bands (*λ*_abs2_) were determined by a peak-fitting program (see Fig. S3 in ESI for detail†). As summarized in Table 1, *λ*_abs2_ and *λ*_abs1_ shifted to shorter wavelengths with increasing ring size. Intense photoluminescence was observed in solutions and their emission wavelength depended, similar to the absorption, on the ring size, *i.e.*, the emission maxima were blue-shifted with increasing size of [*n*]CN. As clearly seen in the CIE coordinates of the [*n*]CNs, variety of the fluorescence colors such as yellow ([8]CN), green ([9]CN), light blue ([10]CN), and deep blue ([12]- and [16]CNs) can be obtained (also see Fig. S2 in ESI†). The absolute fluorescence quantum yields (*Φ*_F_) of the CNs in CH_*s*_Cl_2_ were measured and the values are summarized in Table 1. Similar to CPPs, the *Φ*_F_ values of the CNs increased with increasing size of the CN.

**Table 1:** Photophysical data for [*n*]CNs^*a*^

[*n*]CN	Absorption	Fluorescence		
	*λ*_abs1_ ^*b*^, *λ*_abs2_ ^*c*^ [nm]	*λ*_em_ ^*d*^ [nm]	*Φ*_F_ ^*e*^	CIE_*x*_, CIE_*y*_
[8]CN	397, 447	570	0.23	0.43, 0.52
[10]CN	381, 424	480	0.37	0.16, 0.33
[12]CN	371, 406	458	0.40	0.14, 0.15
[16]CN	350, 378	442	0.43	0.15, 0.07
[9]CN^12^	378, 431	491	0.35	0.19, 0.46

^*a*^In CH_2_Cl_2_.

^*b*^The highest absorption maxima.

^*c*^The longest absorption maxima determined by a peak separation method.

^*d*^Emission maxima upon excitation at *λ*_abs1_.

^*e*^Absolute fluorescence quantum yields determined by a calibrated integrating sphere system within 3% error.

**Fig. 5:**
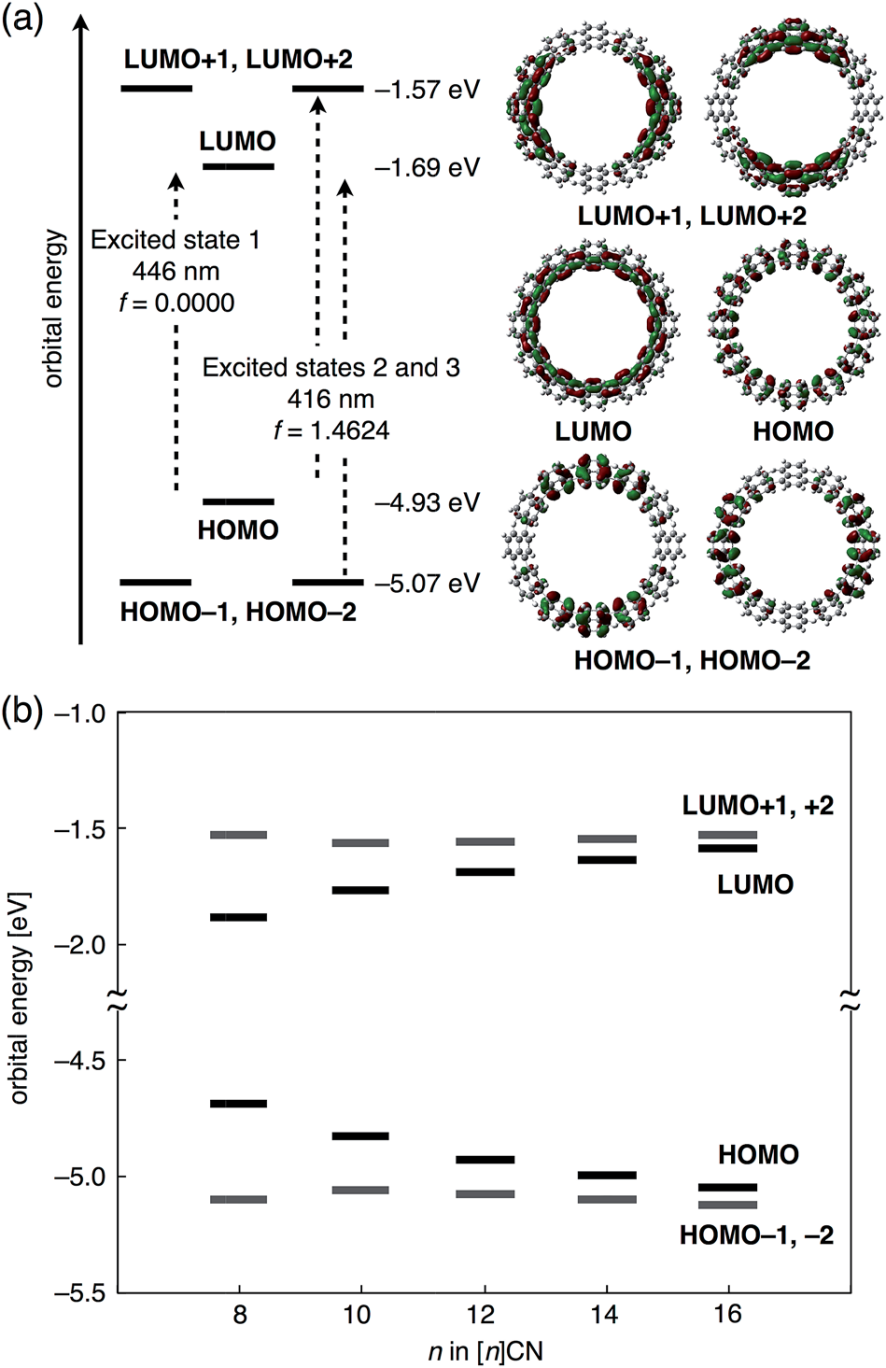
(a) Energy diagrams and pictorial representations of the frontier molecular orbitals for [12]CN calculated at B3LYP/6-31G(d) level of theory. Excitation energies were calculated using TD-DFT at the same level. (b) Frontier orbital energies of [*n*]CNs (*n* = 8, 10, 12, 14, 16).

To investigate the origin of the size-dependency of the photophysical properties of CNs, time-dependent (TD) DFT calculations were carried out on [*n*]CNs (*n* = 8, 10, 12, 14, and 16) at the B3LYP/6-31G(d) level of theory. For all these CNs, similar orbital shapes, as well as degeneracy and transitions were observed. The energy diagrams of the six frontier molecular orbitals (HOMO–2 to LUMO+2) and a pictorial representations of these six orbitals for [12]CN are shown in Fig. 5a. Whereas the HOMO and LUMO of [12]CN are delocalized over the entire ring, the HOMO–1, HOMO–2, LUMO+1, and LUMO+2 are delocalized albeit separated into two sections on opposing sides of the rings. The associated energy diagram reflects the degeneracy for the HOMO–1/HOMO–2 (–5.07 eV) and LUMO+1/LUMO+2 (–1.57 eV) pairs. Considering the shape of the orbitals, the occupied and unoccupied orbitals should represent the π and π* frontier orbitals of the conjugated poly-1,4-naphthylenes. The TD DFT calculations indicated that two characteristic energetically low-lying transitions originate from the set of six orbitals: one transition is a forbidden HOMO → LUMO transition with an oscillator strength (*f*) of 0.00 (excited state 1), while the other is a degenerate transition, in which both the HOMO–1 → LUMO and HOMO → LUMO+1 excitations are mixed with a high *f* value of 1.46 for [12]CN (excited states 2 and 3). All these transitions are π–π* transitions. In the absorption spectrum of [12]CN, the excited states 2 and 3 should correspond to *λ*_abs1_, while the shoulder peak *λ*_abs2_ should correspond to excited state 1. The forbidden transition may be due to a deformation away from high symmetry on account of a dynamic conformational change. Fig. 5b shows the molecular orbital energies for [8]–[16]CNs. With increasing size of the [*n*]CNs, the HOMO levels increased and the LUMO level decreased, whereas the energies of the HOMO–1 and LUMO+1 levels remained largely unaffected. This size-dependency is very similar to those of CPPs.^7^ The origin of this behavior may be ascribed to two factors: (1) the lack of energy dependence of the frontier molecular orbitals on the conjugation length, and (2) the substantial effect of bending and twisting of the naphthalene rings on the orbital energies, as in the case of CPPs.^7*a*^

### Kinetic study on the conformers of [10]CN

In our previous theoretical examination of [9]CN, we predicted that the rotation of the naphthalene rings in [9]CN was affected by the ring strain. However, experimental information on the dynamic behavior of [*n*]CNs had remained elusive. Herein, we successfully obtained a conformational isomer of [10]CN during the synthetic study, which allowed us to determine the isomerization barrier of [10]CN experimentally for the first time.

**Scheme 2:**
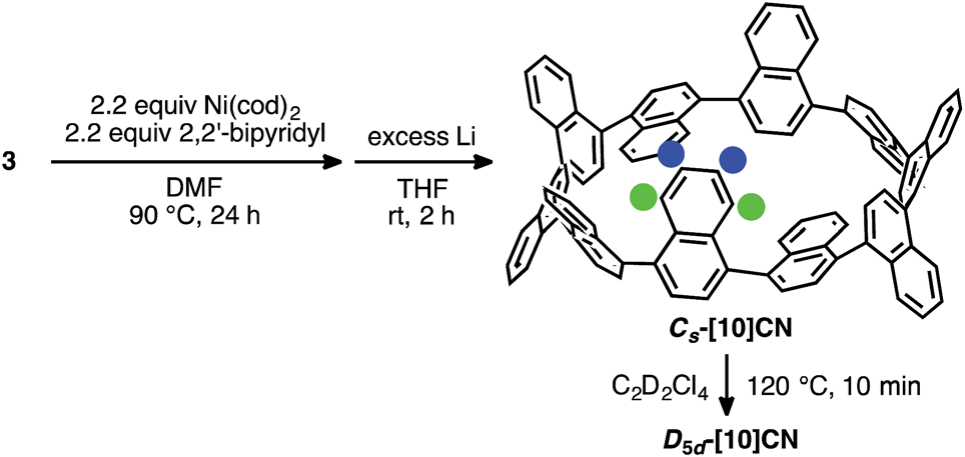
Synthesis of ***C*_s_-[10]CN** and thermal conversion into ***D*_5d_-[10]CN**.

The synthesis of this [10]CN isomer is shown in Scheme 2. When the mixture containing cyclic dimer of **3** was reduced for 2 h less than under optimized conditions (12 hours), a ^1^H NMR spectrum of lower symmetry, including two high-field shifted doublets of doublets at 6.29 ppm and 6.53 ppm, was observed. Considering that this product showed the same mass number as the highly symmetric [10]CN (***D*_5d_-[10]CN**) and that it was converted into ***D*_5d_-[10]CN** upon heating at 120 °C, the product should be a conformer of [10]CN. Supported by the simulation of the ^1^H NMR chemical shift by DFT calculations at the GIAO B3LYP/6-311+G(2d,p)//B3LYP/6-31G(d) level of theory (see ESI for details†), we assumed that this isomer might be ***C*_s_-[10]CN**, in which one naphthalene ring is oriented towards the inside of the CN ring. The high-field shift of the two protons marked by blue and green circles in Scheme 2 might thus be due to the shielding effect of the ring current of the neighboring naphthalene rings.

**Fig. 6:**
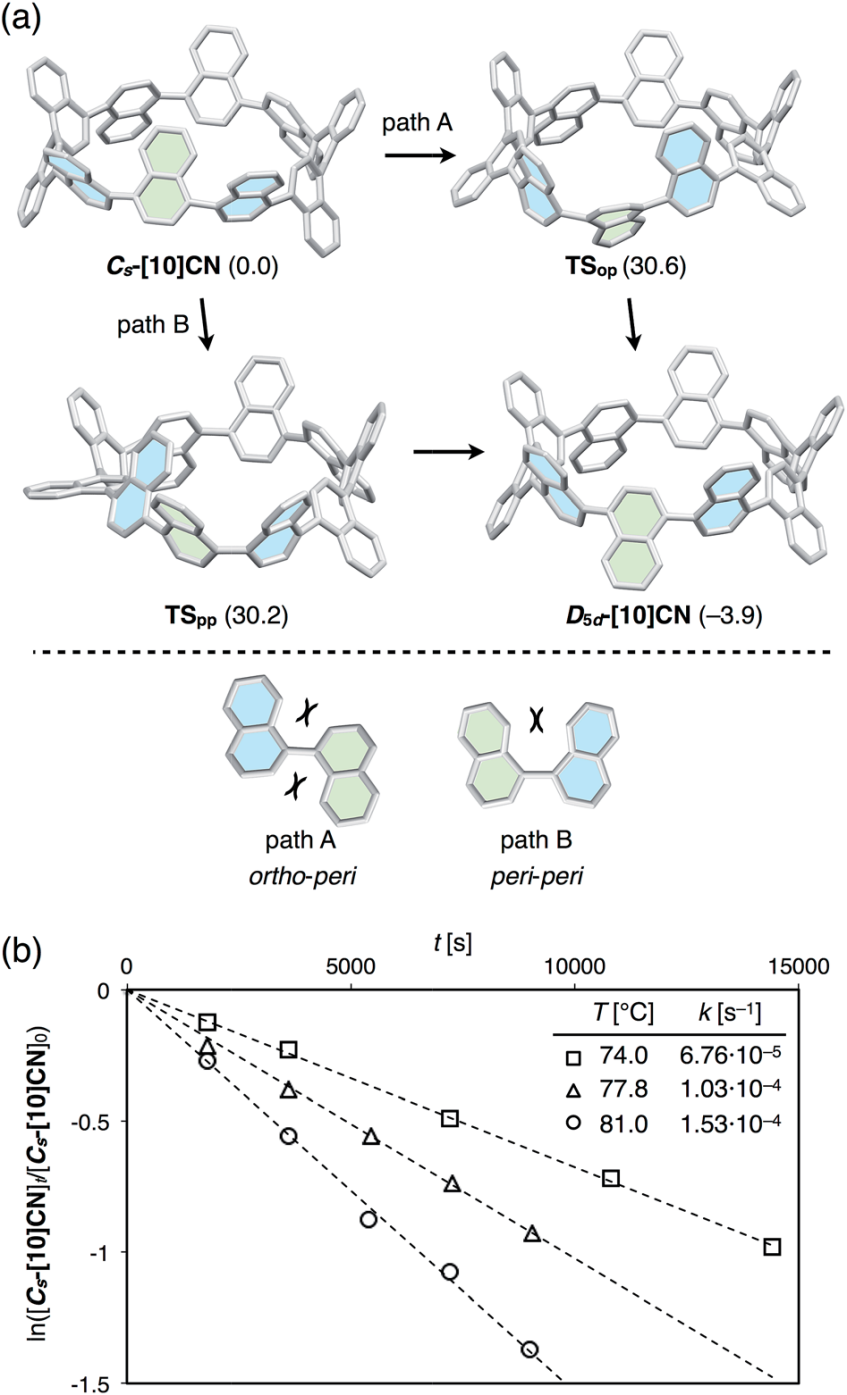
(a) Representative transition states (**TS_op_**, **TS_pp_**) for the two rotation pathways (*ortho*–*peri* and *peri*–*peri* modes) for the conversion of ***C*_s_-[10]CN** into ***D*_5d_-[10]CN**. Calculated Δ*G* values are given in parentheses (kcal mol^–1^) relative to that of ***C*_s_-[10]CN** calculated at B3LYP/6-31G(d) level of theory. (b) Plots for the decreasing integration of ***C*_s_-[10]CN** in the ^1^H NMR spectra in C_2_D_2_Cl_4_ upon heating at 74.0, 77.8, and 81.0 °C, using coronene as the internal standard.

The dynamics of the isomerization from ***C*_s_-[10]CN** to ***D*_5d_-[10]CN** was studied theoretically and experimentally. Initially, we calculated the isomerization pathway from ***C*_s_-[10]CN** to ***D*_5d_-[10]CN**. As shown in Fig. 6a (bottom), two rotation modes are possible for 1,1′-binaphthyl, *i.e. ortho*–*peri* and *peri*–*peri*. For the isomerization of ***C*_s_-[10]CN**, we also found two pathways, corresponding to the *ortho*–*peri* (path A) and *peri*–*peri* mode (path B). For each pathway one intermediate and two transition states were found, and the highest transition state for each pathway is shown in Fig. 6a. Isomerization barriers of 30.6 and 30.2 kcal mol^–1^ were calculated for path A and path B, respectively. Considering that the rotation *via* the *ortho*–*peri* mode is much more favorable for 1,1′-binaphthyl (25.4 and 34.3 kcal mol^–1^ for *ortho*–*peri* and *peri*–*peri*, respectively),^12,19^ the observed destabilization of the *ortho*–*peri* mode and the stabilization of the *peri*–*peri* mode in [10]CN may be due to the ring strain in [10]CN. Although it is difficult to determine which pathway is the favorable isomerization pathway from ***C*_s_-[10]CN** to ***D*_5d_-[10]CN**, we expect that the isomerization should occur under mild heating conditions. Accordingly, it should be possible to determine the isomerization barrier by monitoring the decreasing integration of ***C*_s_-[10]CN** in the ^1^H NMR spectra in 1,1,2,2-tetrachloroethane-*d*_2_ using coronene as the internal standard. The first-order rate constants *k* (s^–1^) of the conversion at various temperatures were estimated using the following equation:ln([***C*_s_-[10]CN**]_*t*_/[***C*_s_-[10]CN**]_0_) = –*kt*wherein [***C*_s_-[10]CN**]_0_ refers to the initial ratio of the integration of ***C*_s_-[10]CN**, whereas [***C*_s_-[10]CN**]_*t*_ refers to the ratio of the integration of ***C*_s_-[10]CN** at time *t* during the conversion (Fig. 6b). The Eyring plot based on these data provided the following activation parameters Δ*H* ^‡^ = 27.7 kcal mol^–1^, Δ*S* ^‡^ = 2.0 cal mol^–1^ K^–1^, and Δ*G*‡298 K = 27.1 kcal mol^–1^. These values are consistent with the theoretically predicted isomerization barriers.

## Conclusions

We have achieved the synthesis of [*n*]CNs (*n* = 8, 10, 12, 16) and uncovered their intriguing structural and photophysical properties. Nickel- or palladium-mediated couplings of the extended L-shaped units **2** and **3**, followed by reductive aromatization of the coupling products afforded [*n*]CNs (*n* = 8, 10, 12, 16). The size-dependent properties of these CNs were examined by UV-vis absorption and fluorescence spectroscopy. Theoretical studies supported a unique influence of the number of naphthalene rings in [*n*]CN on its structural and photophysical properties. A kinetic study on the thermal conversion of the *C*_s_-symmetric conformer of [10]CN (***C*_s_-[10]CN**) into the most stable *D*_5d_-symmetric conformer (***D*_5d_-[10]CN**) indicated that the ring strain substantially affects the rotation barrier of the naphthalene rings in [10]CN. With this series of CNs in hand, further attempts to synthesize carbon nanobelts from CNs *via* sequential cyclodehydrogenation reactions are currently ongoing in our laboratory.
