# Supplementary material for: Synthesis and properties of [8]-, [10]-, [12]-, and [16]cyclo-1,4-naphthylenes
Source: Chem Sci. 2016 Sep 12;8(1):661–7. doi: 10.1039/c6sc04048a (PMC5297897; doi:10.1039/c6sc04048a)

Electronic Supplementary Material (ESI) for Chemical Science.
This journal is © The Royal Society of Chemistry 2016

Supporting Information

Synthesis and Properties of [8]-, [10]-, [12]-, and [16]Cyclo-1,4-naphthylenes

Keishu Okada,[a,‡] Akiko Yagi,[a,‡] Yasutomo Segawa,*[,a,b] and Kenichiro Itami*[,a,b,c]

[a]*Graduate School of Science, Nagoya University, Japan*

[b]*JST, ERATO, Itami Molecular Nanocarbon Project, Nagoya University, Japan.*

[c]*Institute of Transformative Bio-Molecules (WPI-ITbM), Nagoya University, Japan*

E-mail: ysegawa@nagoya-u.jp (Y.S.), itami@chem.nagoya-u.ac.jp (K.I.)

[‡]These authors contributed equally.

Table of Contents

1. Experimental section S2–S10
2. Photophysical study S11–S12
3. Computational study S13–S21
4. Kinetic study S22–S23
5. References S24
6. ^{1}H and ^{13}C NMR spectra of products S25–S40

Experimental section

General

Unless otherwise noted, all materials including dry solvents (1,4-dioxane and *N,N'*-dimethylformamide) were obtained from commercial suppliers and used without further purification. Tetrahydrofuran (THF) and toluene were purified by passing through a solvent purification system (Glass Contour). All reactions were performed with dry solvents under an atmosphere of nitrogen in dried glassware with standard vacuum-line techniques. Work-up and purification procedures were carried out with reagent-grade solvents under air unless otherwise noted. 1,4-Dibromonaphthalene[1] and **1a**[2] were prepared according to procedures reported in the literatures.

Analytical thin-layer chromatography (TLC) was performed using E. Merck silica gel 60 F_{254} precoated plates (0.25 mm). The developed chromatogram was analyzed by UV lamp (254 nm and 365 nm). Flash column chromatography was performed with E.Merck silica gel 60 (230–400 mesh). Preparative thin-layer chromatography (PTLC) was performed using Wako-gel® B5-F silica coated plates (0.75 mm) prepared in our laboratory. Preparative gel permeation chromatography (GPC) was performed with a JAI LC-9204 instrument equipped with JAIGEL-2H/JAIGEL-2H columns using chloroform as an eluent. High-resolution mass spectra (HRMS) were obtained from a JEOL JMS700 (fast atom bombardment mass spectrometry, FAB MS) or a JEOL JMS-S3000 SpiralTOF (MALDI-TOF MS) with *trans*-2-[3-(4-tert-butylphenyl)-2-methyl-2-propenylidene]malononitrile as matrix. Nuclear magnetic resonance (NMR) spectra were recorded on a JEOL JNM-ECA-600 (^{1}H 600 MHz, ^{13}C 150 MHz) spectrometer. Chemical shifts for ^{1}H NMR are expressed in parts per million (ppm) relative to $CHCl_3$ (δ 7.26 ppm), C_2DHCl_4 (δ 5.98 ppm) or $CHDCl_2$ (δ 5.32 ppm). Chemical shifts for ^{13}C NMR are expressed in ppm relative to $CDCl_3$ (δ 77.0 ppm), C_2DHCl_4 (δ 5.98 ppm) or CD_2Cl_2 (δ 53.8 ppm). Data are reported as follows: chemical shift, multiplicity (s = singlet, d = doublet, dd = doublet of doublets, ddd = doublet of doublets of doublets, t = triplet, m = multiplet, br = broad signal), coupling constant (Hz), and integration.

UV-vis absorption spectra of CNs were recorded on a Shimadzu UV-3510 spectrometer with a resolution of 0.5 nm. Emission spectra of CNs was measured with an F-4500 Hitachi spectrometer with a resolution of 0.4 nm upon excitation at each absorption maximum wavelength. Dilute solution in degassed spectral grade dichloromethane in 1 cm square quartz cell was used for measurements. Absolute fluorescence quantum yield was determined with a Hamamatsu C9920-02 calibrated integrating sphere system upon excitation at each absorption maximum wavelength.

Synthesis of 1b

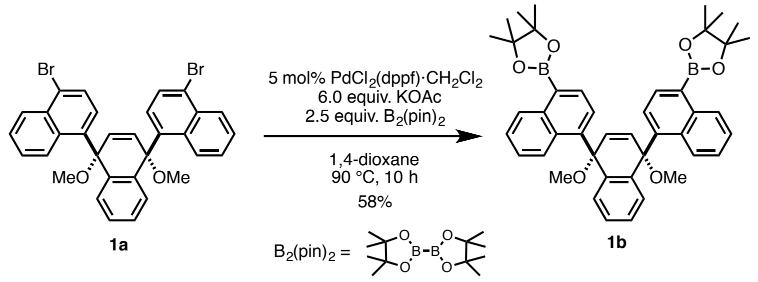

To a two-necked 100 mL glass round bottom flask containing a magnetic stirring bar were added **1a** (2.50 g, 4.16 mmol), $PdCl_2(dppf)\cdot CH_2Cl_2$ (170 mg, 208 μmol), dried KOAc (2.45 g, 25.0 mmol), and bis(pinacolato)diboron (2.67 g, 10.4 mmol), and the flask was evacuated and filled with argon three times. Dry 1,4-dioxane (42 mL) was added via syringe. After stirring at 90 °C for 10 h, the mixture was passed through a short pad of silica gel (EtOAc) and the solvent was removed under reduced pressure. The crude product was subjected to GPC to afford **1b** (1.68 g, 58%) as a white solid.

^{1}H NMR (600 MHz, $CDCl_3$) δ 1.39 (s, 12H), 1.40 (s, 12H), 3.36 (s, 6H), 6.74 (s, 2H), 7.15 (brs, 2H), 7.31–7.42 (m, 4H), 7.45 (ddd, *J* = 8.4, 6.6, 1.2 Hz, 2H), 7.56 (dd, *J* = 6.0, 3.6 Hz, 2H), 7.88 (d, *J* = 7.2 Hz, 2H), 8.75 (dd, *J* = 7.2, 0.9 Hz, 2H), 8.81 (brs, 2H); ^{13}C NMR (150 MHz, CD_2Cl_2) δ 25.01 (CH_3), 25.04 (CH_3), 25.08 (CH_3), 25.11 (CH_3), 51.6 (CH_3), 79.8 (4°), 84.2 (4°), 125.2 (CH), 125.7 (CH), 126.3 (CH), 127.7 (CH), 128.7 (CH), 129.4 (CH), 129.6 (CH), 131.6 (4°), 132.9 (CH), 134.6 (CH), 138.6 (4°), 139.5 (4°), 143.5 (4°); HRMS (FAB) *m/z* calcd. for $C_{44}H_{48}NaO_6B_2$ $[M\cdot Na]^+$: 717.3535, found: 717.3539.

Synthesis of 2

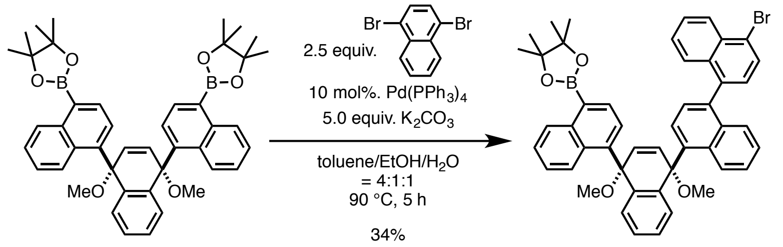

1b **2**

To a two-necked 100 mL glass round bottom flask containing a magnetic stirring bar were added **1b** (400 mg, 576 μmol), K_2CO_3 (398 mg, 2.88 mmol), and 1,4-dibromonaphthalene (416 mg, 1.44 mmol), and the flask was evacuated and filled with argon three times. Dry toluene (29 mL) was added via syringe. Subsequently, degassed EtOH (7 mL) and degassed distilled water (7 mL) were added. Then, $Pd(PPh_3)_4$ (66.6 mg, 57.6 μmol) was added under argon flow, and the mixture was stirred at 90 °C for 5 h. After the reaction mixture was cooled to room temperature, brine (ca. 50 mL) was added to the mixture. The mixture was extracted with EtOAc (50 mL × 3). The combined organic phase was then purified by silica chromatography (hexane/EtOAc = 30:1→20:1) and GPC to afford **2** (150 mg, 34%) as a white solid.

^{1}H NMR (600 MHz, CD_2Cl_2) δ 1.33 (s, 6H), 1.34 (s, 6H), 1.37 (s, 12H), 3.36 (s, 6H), 3.41 (s, 6H), 6.76 (d, J = 10.5 Hz, 1H), 6.78 (d, J = 10.5 Hz, 1H), 6.92 (d, J = 10.5 Hz, 1H), 6.94 (d, J = 10.5 Hz, 1H), 7.12 (d, J = 7.4 Hz, 1H), 7.15 (d, J = 7.4 Hz, 1H), 7.18 (d, J = 7.7 Hz, 1H) 7.21–7.64 (m, 28H) 7.73 (d, J = 7.9 Hz, 1H), 7.78 (d, J = 9.3 Hz, 1H), 7.79 (d, J = 7.2 Hz, 1H), 7.80 (d, J = 7.3 Hz, 1H), 7.87 (d, J = 7.6 Hz, 1H), 7.90 (d, J = 7.6 Hz, 1H), 8.32 (d, J = 6.2 Hz, 1H) 8.33 (d, J = 8.4 Hz, 1H), 8.74 (d, J = 8.5 Hz, 2H), 8.85 (d, J = 7.9 Hz, 2H), 9.02 (d, J = 8.4 Hz, 1H); ^{13}C NMR (150 MHz, CD_2Cl_2) δ 24.98 (CH_3), 25.02 (CH_3), 25.1 (CH_3), 51.64 (CH_3), 51.63 (CH_3), 51.79 (CH_3), 51.83 (CH_3), 79.59 (4°), 79.65 (4°), 80.0 (4°) 80.1 (4°), 84.2 (4°), 84.2 (4°), 122.8 (CH), 125.3 (CH), 125.65 (CH), 125.69 (CH), 125.74 (CH), 125.77 (CH), 125.78 (CH), 125.83 (CH), 126.18 (CH), 126.24 (CH), 126.86 (CH), 126.93 (CH), 127.17 (CH), 127.24 (CH), 127.56 (CH), 127.63 (CH), 127.66 (CH), 127.71 (CH), 127.9 (CH), 128.0 (CH), 128.5 (CH), 128.76 (CH), 128.81 (CH), 128.85 (CH), 128.88 (CH), 129.42 (CH), 129.45 (CH), 129.5 (CH), 129.60 (CH), 129.64 (CH), 129.9 (CH), 131.4 (CH), 131.5 (CH), 132.1 (CH), 132.2 (CH), 133.0 (CH), 133.09 (CH), 133.13 (CH), 133.2 (CH), 134.3 (CH), 134.4 (CH), 134.5 (CH), 134.7 (CH), 138.49 (CH), 138.54 (CH), 138.6 (CH), 139.1 (CH), 139.3 (CH), 139.4 (CH), 139.79 (CH), 139.80 (CH), 140.5 (CH), 140.6 (CH), 143.55 (CH), 143.57 (CH); HRMS (FAB) m/z calcd. for $C_{48}H_{42}BrO_4BNa$ $[M{\cdot}Na]^+$: 795.2257, found: 795.2232.

Synthesis of 3

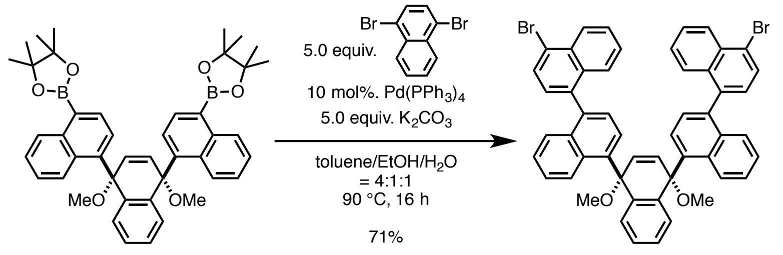

To a two-necked 200-mL glass round bottom flask containing a magnetic stirring bar were added **1b** (802 mg, 1.15 mmol), K_2CO_3 (796 mg, 5.76 mmol), and 1,4-dibromonaphthalene (1.67 g, 5.76 mmol), and the flask was evacuated and filled with argon three times. Dry toluene (38 mL) was added via syringe. Subsequently, degassed EtOH (9 mL) and degassed distilled water (9 mL) were added. Then, $Pd(PPh_3)_4$ (133 mg, 115 μmol) was added under argon flow, and the mixture was stirred at 90 °C for 16 h. After the reaction mixture was cooled to room temperature, brine (ca. 100 mL) was added to the mixture. The mixture was extracted with EtOAc (100 mL × 3). The combined organic phase was then purified by silica chromatography (hexane/EtOAc = 20:1) and GPC to afford **3** (633 mg, 71%) as a white solid.

^{1}H NMR (600 MHz, CD_2Cl_2) δ 3.427 (s, 3H), 3.430 (s, 6H), 3.44 (s, 3H), 6.95 (d, J = 6.0 Hz, 4H), 7.06 (t, J = 7.2 Hz, 1H), 7.14 (d, J = 7.8 Hz, 1H), 7.16–7.25 (m, 14H), 7.27–7.34 (m, 11H), 7.36–7.45 (m, 4H), 7.48 (t, J = 7.2 Hz, 1H), 7.51–7.60 (m, 8H), 7.72–7.82 (m, 5H), 7.859 (d, J = 7.2 Hz, 1H), 7.861 (d, J = 7.8 Hz, 1H), 7.88 (d, J = 7.8 Hz, 1H), 8.28 (d, J = 8.4 Hz, 1H), 8.29 (d, J = 9.0 Hz, 1H), 8.31 (d, J = 8.4 Hz, 1H), 8.32 (d, J = 8.5 Hz, 1H), 8.98 (d, J = 8.4 Hz, 1H), 9.00 (d, J = 8.4 Hz, 1H), 9.01(d, J = 6.5 Hz, 1H), 9.02 (d, J = 8.4 Hz, 1H); ^{13}C NMR (150 MHz, CD_2Cl_2) δ 51.78 (CH_3), 51.79 (CH_3), 79.8 (4°), 79.9 (4°), 79.96 (4°), 80.00 (4°), 122.77 (CH), 122.79 (CH), 122.81 (CH), 125.67 (CH), 125.72 (CH), 125.76 (CH), 125.78 (CH), 125.83 (CH), 126.8 (CH), 126.9 (CH), 127.0 (CH), 127.1 (CH), 127.3 (CH), 127.5 (CH), 127.55 (CH), 127.61 (CH), 127.70 (CH), 127.72 (CH), 127.8 (CH), 127.87 (CH), 127.91 (CH), 127.94 (CH), 128.48 (CH), 128.50 (CH), 128.6 (CH), 128.90 (CH), 128.92 (CH), 129.0 (CH), 129.5 (CH), 129.59 (CH), 129.61 (CH), 129.8 (CH), 129.85 (CH), 129.89 (CH), 129.94 (CH), 131.98 (CH), 132.02 (CH), 132.1 (CH), 132.2 (CH), 133.1 (CH), 133.2 (CH), 133.35 (CH), 133.38 (CH), 134.38 (CH), 134.40 (CH), 134.44 (CH), 138.5 (CH), 138.56 (CH), 138.59 (CH), 139.06 (CH), 139.08 (CH), 139.14 (CH), 139.6 (CH), 139.68 (CH), 139.72 (CH), 140.55 (CH), 140.60 (CH), 140.7 (CH); HRMS (FAB) m/z calcd. for $C_{52}H_{36}Br_2O_2Na$ $[M{\cdot}Na]^+$: 873.0980, found: 873.0966.

Synthesis of [8]-, [12]-, and [16]CN

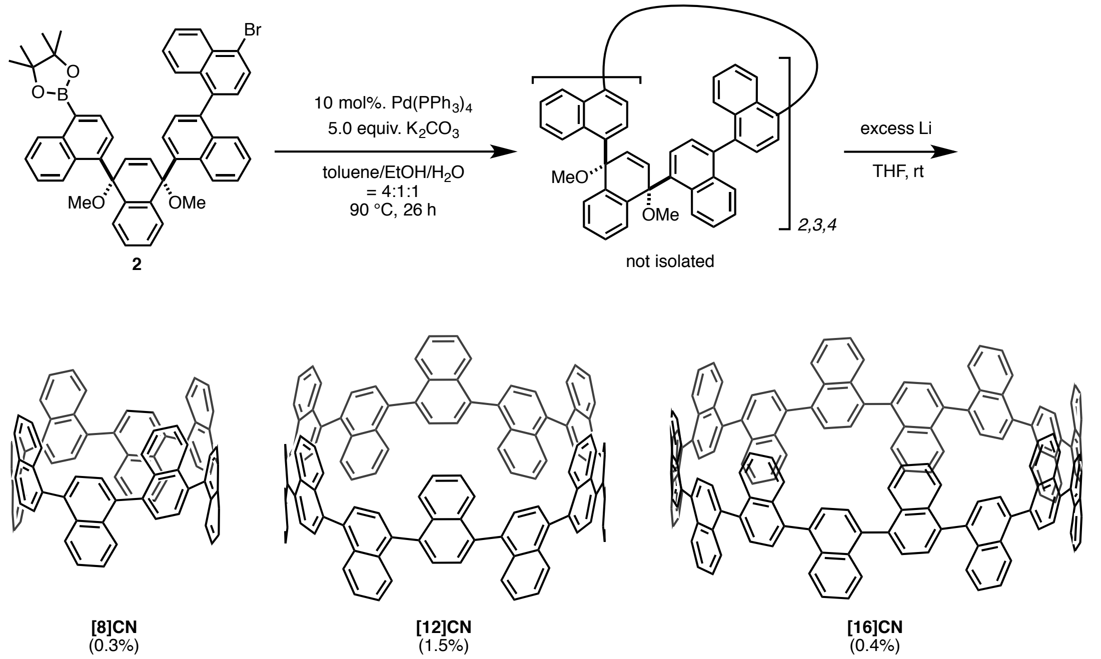

To a two-necked 500 mL glass round bottom flask containing a magnetic stirring bar was added **2** (580 mg, 750 μmol), and the flask was evacuated and filled with argon three times. Dry toluene (290 mL) was added via syringe. Subsequently, K_2CO_3 (518 mg, 3.75 mmol), degassed EtOH (70 mL) and degassed distilled water (70 mL) were added. Then, $Pd(PPh_3)_4$ (86.6 mg, 75.0 μmol) was added under argon flow, and the mixture was stirred at 90 °C for 26 h. After the reaction mixture was cooled to room temperature, brine (ca. 200 mL) was added to the mixture. The mixture was extracted with EtOAc (200 mL × 3). The combined organic phase was then subjected to GPC. The mixture containing macrocycles were subjected to the next aromatization step directly.

A 30-mL vials containing glass-coated magnetic stirring bars and cyclization products were dried under vacuum and filled with argon after cooling to room temperature. In a glove box, lithium granular (20–30 mg, 2.9–4.3 mmol) and dry THF (3 mL) were added to the vials. The reaction mixtures were stirred at room temperature for 8–12 h. The residues were diluted with hexane and quenched with methanol. After evaporated, the reaction mixtures were passed through a short silica gel pad (CH_2Cl_2). The filtrates were evaporated and purified by PTLC (CH_2Cl_2/hexane = 1:1) to obtain **[8]CN** (1.5 mg containing hydrocarbon solvents, 0.1% from **2**) as an orange solid, **[12]CN** (5.7 mg, 1.5% from **2**) as a yellow solid, and **[16]CN** (1.6 mg, 0.4% from **2**) as a white solid.

[8]CN: ^{1}H NMR (600 MHz, CD_2Cl_2) δ 6.98 (s, 16H), 7.59 (dd, J = 6.6, 3.6 Hz, 16H), 8.52 (dd, J = 6.6, 3.6 Hz, 16H); ^{13}C NMR (150 MHz, CD_2Cl_2) δ 126.7 (CH), 127.3 (CH), 129.4 (CH), 134.0 (4°), 138.6 (4°). HRMS (MALDI-TOF MS) m/z calcd. for $C_{80}H_{48}$ $[M]^+$: 1008.3756, found: 1008.3763.

[12]CN: ^{1}H NMR (600 MHz, CD_2Cl_2) δ 7.27 (s, 24H), 7.61 (dd, J = 6.5, 3.4 Hz, 24H), 8.48 (dd, J = 6.5, 3.4 Hz, 24H); ^{13}C NMR (150 MHz, CD_2Cl_2) δ 126.6 (CH), 127.2 (CH), 129.2 (CH), 134.2 (4°),

138.4 (4°). HRMS (MALDI-TOF MS) *m/z* calcd. for $C_{120}H_{72}$ $[M]^+$: 1512.5634, found: 1512.5649.

[16]CN: ^{1}H NMR (600 MHz, CD_2Cl_2) δ 7.43 (s, 32H), 7.61 (dd, *J* = 6.6, 3.3 Hz, 32H), 8.34 (dd, *J* = 6.0, 3.3 Hz, 32H); ^{13}C NMR (150 MHz, CD_2Cl_2) δ 126.6 (CH), 127.3 (CH), 129.1 (CH), 134.1 (4°), 138.7 (4°). HRMS (MALDI-TOF MS) *m/z* calcd. for $C_{160}H_{96}$ $[M]^+$: 2016.7512, found: 2016.7523.

Synthesis of D_{5d}-[10]CN

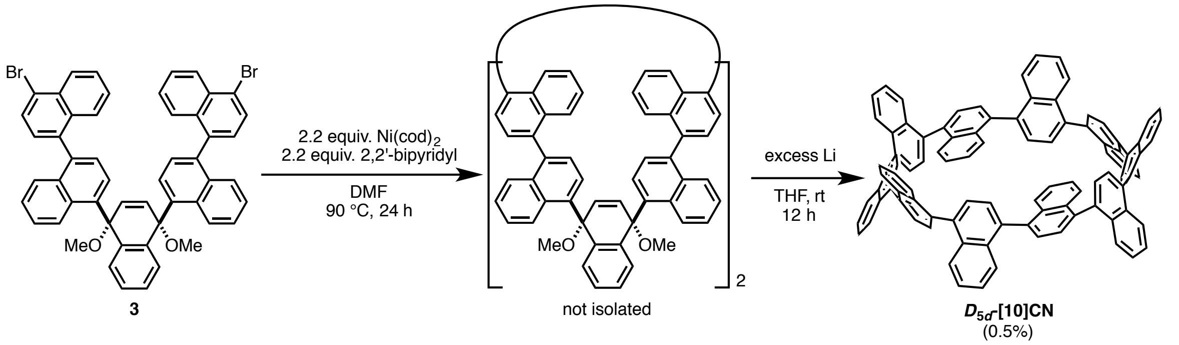

(Batch A) To a two-necked 300 mL glass round bottom flask containing a magnetic stirring bar were added **3** (150 mg, 176 μmol) and 2,2'-bipyridyl (60.5 mg, 387 μmol), then the flask was evacuated and filled with argon three times. $Ni(cod)_2$ (107 mg, 387 μmol) was added to the flask in a glove box. Subsequently, dry *N,N'*-dimethylformamide (150 mL) were added via syringe. Then, the mixture were stirred at 90 °C for 24 h. After the reaction mixtures were cooled to room temperature, brine (ca. 200 mL) was added.

(Batch B) To a two-necked 300 mL glass round bottom flask containing a magnetic stirring bar were added **3** (120 mg, 141 μmol) and 2,2'-bipyridyl (48.4 mg, 310 μmol), then the flask was evacuated and filled with argon three times. $Ni(cod)_2$ (85.1 mg, 310 μmol) was added to the flask in a glove box. Subsequently, dry *N,N'*-dimethylformamide (150 mL) were added via syringe. Then, the mixture were stirred at 90 °C for 24 h. After the reaction mixtures were cooled to room temperature, brine (ca. 200 mL) was added.

The two reaction mixtures (batches A and B) were combined and extracted with EtOAc (400 mL × 3). The combined organic phase was subjected to GPC and then by PTLC (CH_2Cl_2/hexane = 2:1). The mixture containing macrocycles were subjected to the next aromatization step directly. A 30-mL vial containing glass-coated magnetic stirring bars and cyclization products was dried under vacuum and filled with argon after cooling to room temperature. In a glove box, lithium granular (20 mg, 2.9 mmol) and dry THF (3 mL) were added to the vial. The reaction mixture was stirred at room temperature for 7 h. The residues were diluted with hexane and quenched with methanol. After evaporated, the reaction mixtures were passed through a short silica gel pad (CH_2Cl_2). The filtrates were evaporated and purified by PTLC (CH_2Cl_2/hexane = 1:1) to obtain **D_{5d}-[10]CN** (1.5 mg, 0.5% from **3**) as a yellow solid.

^{1}H NMR (600 MHz, CD_2Cl_2) δ 7.14 (s, 20H), 7.61 (dd, J = 6.5, 3.4 Hz, 20H), 8.51 (dd, J = 6.5, 3.4 Hz, 20H); ^{13}C NMR (150 MHz, CD_2Cl_2) δ 126.7 (CH), 127.3 (CH), 129.3 (CH), 134.2 (4°), 138.5 (4°). HRMS (MALDI-TOF MS) m/z calcd. for $C_{100}H_{60}$ $[M]^+$: 1260.4695, found: 1260.4678.

Synthesis of C_s-[10]CN

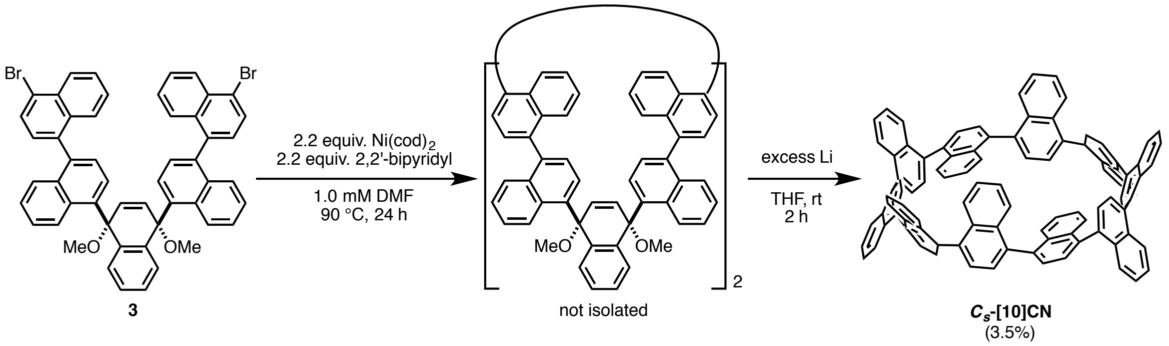

(Batches A and B) To a two-necked 300 mL glass round bottom flask containing a magnetic stirring bar were added **3** (150 mg, 176 μmol) and 2,2'-bipyridyl (60.5 mg, 387 μmol), then the flask was evacuated and backfilled with argon three times. In a glove box, Ni(cod)$_2$ (107 mg, 387 μmol) was added to the flask. Subsequently, dry *N,N'*-dimethylformamide (150 mL) were added via syringe. Then, the mixture was stirred at 90 °C for 24 h. After the reaction mixtures were cooled to room temperature, brine (ca. 200 mL) was added.

(Batch C) To a two-necked 300 mL glass round bottom flask containing a magnetic stirring bar were added **3** (120 mg, 141 μmol) and 2,2'-bipyridyl (48.4 mg, 310 μmol), then the flask was evacuated and backfilled with argon three times. In a glove box, Ni(cod)$_2$ (85.1 mg, 310 μmol) was added to the flask. Subsequently, dry *N,N'*-dimethylformamide (150 mL) were added via syringe. Then, the mixture was stirred at 90 °C for 24 h. After the reaction mixtures were cooled to room temperature, brine (ca. 200 mL) was added.

The three reaction mixtures (batches A, B, and C) were combined and extracted with EtOAc (400 mL × 3). The combined organic phase was then purified by GPC. The purified products were subjected to the next aromatization step directly. A 30-mL vial containing glass-coated magnetic stirring bars and cyclization products was dried under vacuum and filled with argon after cooling to room temperature. In a glove box, lithium granular (20 mg, 2.9 mmol) and dry THF (3 mL) were added to the vial. The reaction mixture was stirred at room temperature for 2 h. The residues were diluted with hexane and quenched with methanol. After evaporated, the reaction mixtures were passed through a short silica gel pad (CH_2Cl_2). The filtrates were evaporated and purified by PTLC (CH_2Cl_2/hexane = 3:2) to obtain ***C$_s$*-[10]CN** (11.0 mg, 3.5%) as a yellow solid.

^{1}H NMR (600 MHz, CD_2Cl_2) δ 6.29 (dd, J = 6.6, 3.6 Hz, 2H), 6.53 (dd, J = 6.6, 3.6 Hz, 2H), 6.89 (d, J = 7.8Hz, 2H) 7.04–7.71 (m, 34H), 7.96 (s, 2H), 8.00 (d, J = 7.8Hz, 2H), 8.45–8,74 (m, 16H); ^{13}C NMR (150 MHz, CD_2Cl_2) δ 125.4 (CH), 125.9 (CH), 126.1 (CH), 126.4 (CH), 126.5 (CH), 126.6 (CH), 126.7 (CH), 126.9 (CH), 127.3 (CH), 127.4 (CH), 127.5 (CH), 127.6 (CH), 127.9 (CH), 128.1 (CH), 128.7 (CH), 129.2 (CH), 129.3 (CH), 129.4 (CH), 129.58 (CH), 129.63 (CH), 129.7 (CH), 129.9 (CH), 130.1 (CH), 130.1 (CH), 130.3 (CH), 131.4 (CH), 132.1 (4°), 132.4 (4°), 132.6 (4°),

133.4 (4°), 133.6 (4°), 133.7 (4°), 134.0 (4°), 134.1 (4°), 134.2 (4°), 134.3 (4°), 134.4 (4°), 134.5 (4°), 135.2 (4°), 135.8 (4°), 138.3 (4°), 138.4 (4°), 138.4 (4°), 138.5 (4°), 138.5 (4°), 138.6 (4°), 138.6 (4°), 138.8 (4°), 139.0 (4°), 139.1 (4°), 139.9 (4°), 140.0 (4°), 140.1 (4°). HRMS (MALDI-TOF MS) *m/z* calcd. for $C_{100}H_{60}$ $[M]^+$: 1260.4695, found: 1260.4702.

2. Photophysical study

UV/Vis absorption spectra were recorded on a Shimadzu UV-3510 spectrometer with a resolution of 0.5 nm. Emission spectra were measured with an FP-6600 Hitachi spectrometer with a resolution of 0.4 nm. Absolute fluorescence quantum yields were determined with a Hamamatsu C9920-02 calibrated integrating sphere system equipped with multichannel spectrometer (PMA-11).

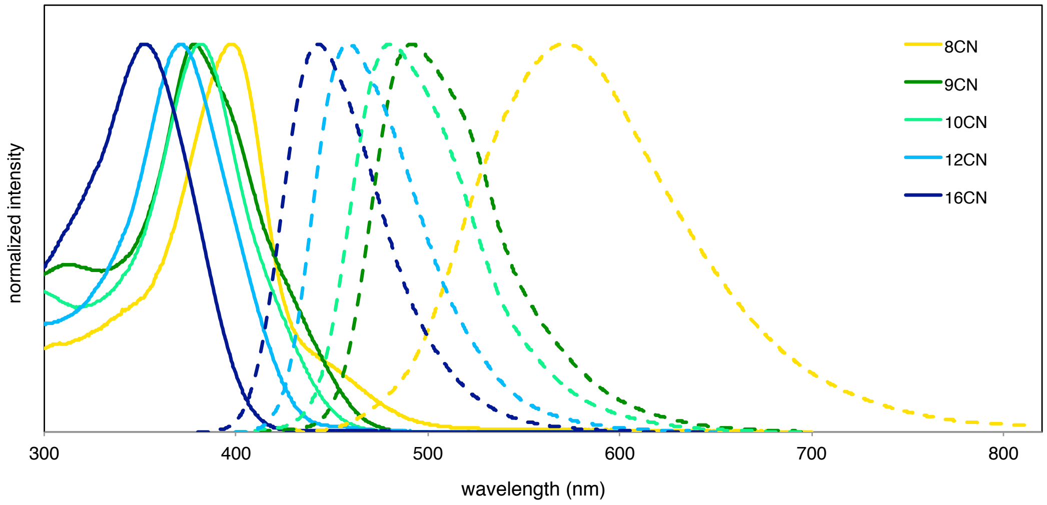

Figure S1. UV–vis absorption (solid line) and fluorescence spectra (broken line) of [8]-, [9]-, [10]-, [12]-, and [16]CNs in dichloromethane.

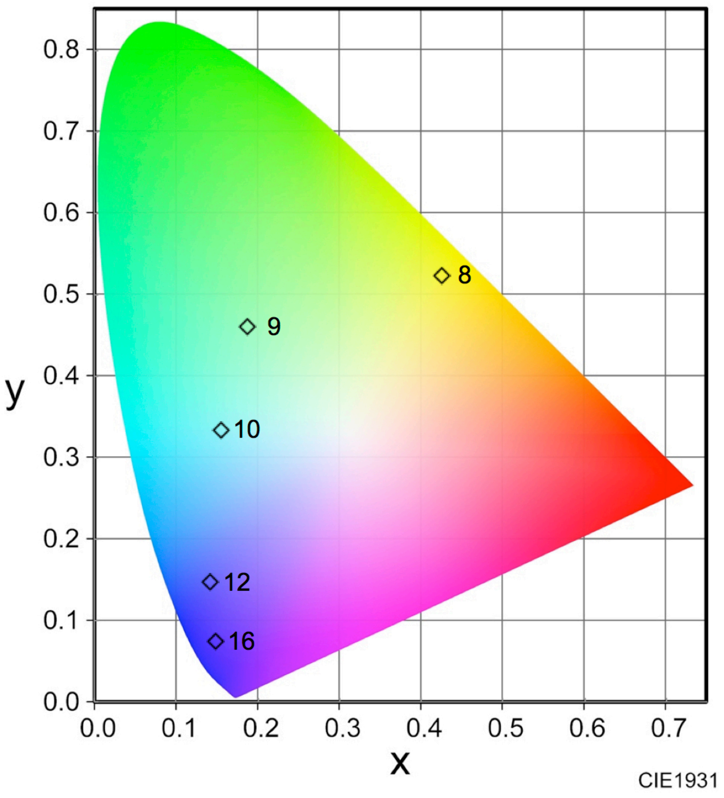

Figure S2. CIE diagram of [*n*]CNs.

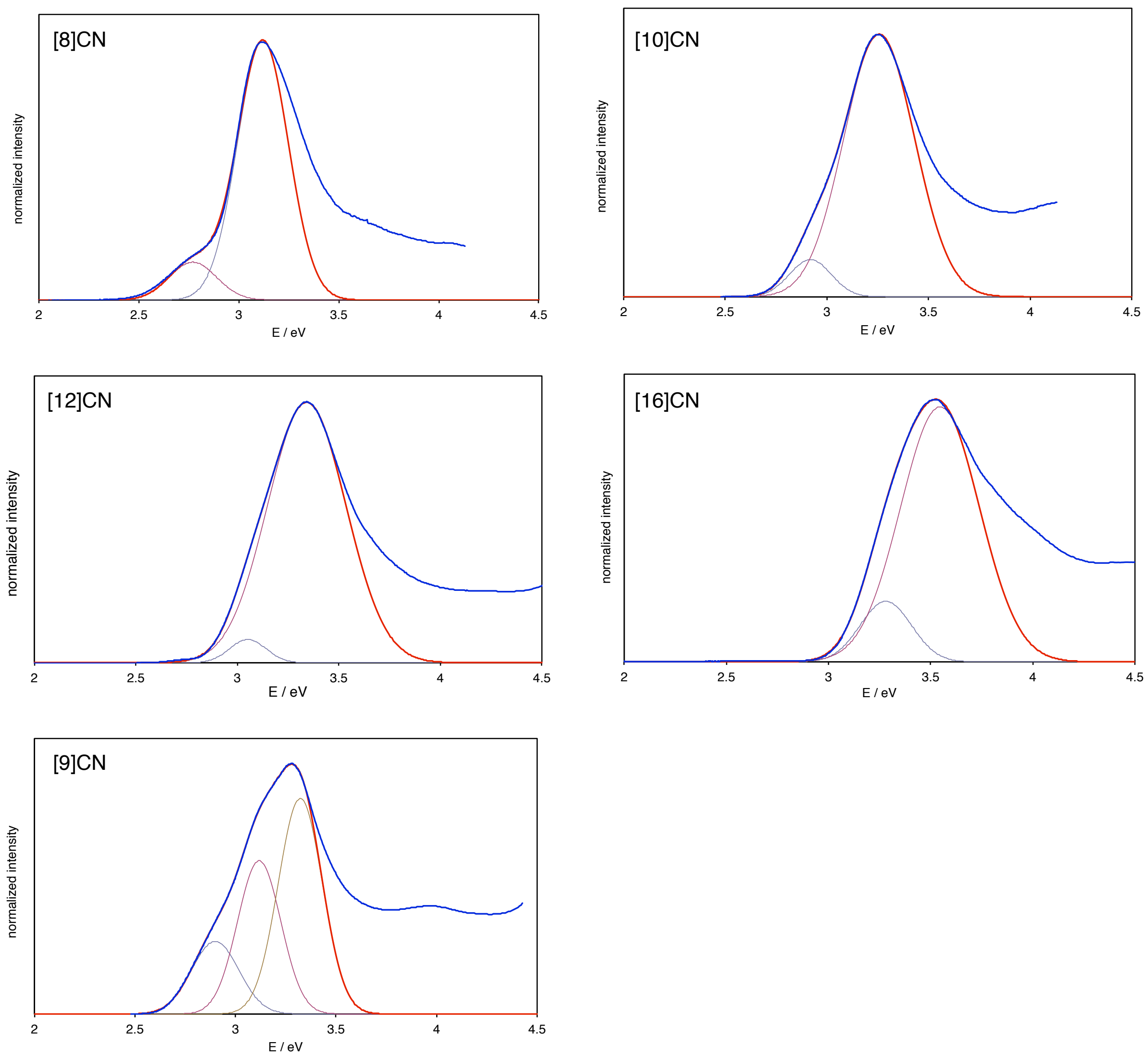

Figure S3. Peak-fitting of the absorption spectra of [*n*]CNs. Blue: observed, red: simulated.

3. Computational study

The Gaussian 09 program[3] running on a SGI Altix4700 system was used for optimization (B3LYP/6-31G(d)[4]). All structures were optimized without any symmetry assumptions. Zero-point energy, enthalpy, and Gibbs free energy at 298.15 K and 1 atm were estimated from the gas-phase studies unless otherwise noted. Harmonic vibration frequency calculations at the same level were performed to verify all stationary points as local minima (with no imaginary frequency) or transition states (with one imaginary frequency). IRC calculations[5] were also performed to check transition states.

Table S1. Strain energies of [*n*]CN and [*n*]CPP.

n	strain energy [kcal·mol^{-1}]	
	[*n*]CN[a]	[*n*]CPP[6]
6	73.2	96.0
7	71.4	84.0
8	50.6	72.2
9	50.4	65.6
10	37.0	57.7
11	36.9	53.7
12	27.8	48.1
13	27.8	45.5
14	21.6	41.0
15	21.4	39.2
16	16.9	35.6

(a) By using the following homodesmotic reactions: [*n*]CN + *n* binaphthyl → *n* ternaphthyl.[2]

Table S2. TD-DFT vertical one-electron excitations (6 states) calculated for [8]CN.

Energy	Wavelength	Oscillator strength (*f*)	Description
2.3244 eV	533.40 nm	0.0000	HOMO -> LUMO (0.69301)
2.8300 eV	438.11 nm	0.2890	HOMO–2 -> LUMO (-0.17133)<br>HOMO -> LUMO+1 (0.67601)
2.8300 eV	438.10 nm	0.2889	HOMO–1 -> LUMO (-0.17144)<br>HOMO -> LUMO+2 (0.67598)
2.9069 eV	426.51 nm	0.7969	HOMO–2 -> LUMO (0.65159)<br>HOMO-1 -> LUMO (-0.23999)<br>HOMO -> LUMO+2 (0.67601)
2.9069 eV	426.51 nm	0.7968	HOMO–2 -> LUMO (0.66975)<br>HOMO-1 -> LUMO (0.10078)<br>HOMO -> LUMO+1 (0.18323)
3.1279 eV	396.38 nm	0.0000	HOMO–2 -> LUMO+1 (-0.20852)<br>HOMO–2 -> LUMO+2 (-0.44951)<br>HOMO–1 -> LUMO+1 (0.44966)<br>HOMO–1 -> LUMO+2 (-0.20848)

Table S3. TD-DFT vertical one-electron excitations (6 states) calculated for [10]CN.

Energy	Wavelength	Oscillator strength (*f*)	Description
2.6021 eV	476.47 nm	0.0000	HOMO–2 ->LUMO+2 (0.17227)<br>HOMO–1 ->LUMO+1 (-0.17257)<br>HOMO -> LUMO (0.66061)
2.9135 eV	425.56 nm	1.2165	HOMO–1 -> LUMO (-0.37106)<br>HOMO -> LUMO+1 (0.59277)
2.9139 eV	425.49 nm	1.2159	HOMO–2 -> LUMO (0.36972)<br>HOMO -> LUMO+2 (0.59352)
3.0020 eV	413.01 nm	0.0488	HOMO–1 -> LUMO (0.59231)<br>HOMO -> LUMO+1 (0.36345)
3.0024 eV	412.95 nm	0.0499	HOMO–2 -> LUMO (0.48729)<br>HOMO -> LUMO+2 (0.48880)
3.1377 eV	395.14 nm	0.0000	HOMO–2 -> LUMO+2 (-0.45757)<br>HOMO–1 -> LUMO+1 (0.46242)<br>HOMO -> LUMO (-0.25046)

Table S4. TD-DFT vertical one-electron excitations (6 states) calculated for [12]CN.

Energy	Wavelength	Oscillator strength (*f*)	Description
2.7819 eV	445.68 nm	0.0000	HOMO–2 ->LUMO+2 (-0.24408)<br>HOMO–1 ->LUMO+1 (-0.24408)<br>HOMO -> LUMO (0.60812)
2.9801 eV	416.04 nm	1.4624	HOMO–1 -> LUMO (-0.43393)<br>HOMO -> LUMO+1 (0.52861)
2.9801 eV	416.04 nm	1.4624	HOMO–2 -> LUMO (-0.43393)<br>HOMO -> LUMO+2 (0.52861)
3.1317 eV	395.90 nm	0.0051	HOMO–1 -> LUMO (0.52236)<br>HOMO -> LUMO+1 (0.41590)<br>HOMO -> LUMO+2 (0.12603)
3.1317 eV	395.90 nm	0.0051	HOMO–1 -> LUMO (0.52236)<br>HOMO -> LUMO+1 (-0.12603)<br>HOMO -> LUMO+2 (0.41590)
3.1840 eV	389.39 nm	0.0000	HOMO–2 -> LUMO+1 (-0.45757)<br>HOMO–1 -> LUMO+2 (0.46242)

Table S5. TD-DFT vertical one-electron excitations (6 states) calculated for [16]CN.

Energy	Wavelength	Oscillator strength (*f*)	Description
2.9992 eV	413.39 nm	0.0000	HOMO -> LUMO (0.50414)<br>HOMO–1 -> LUMO+1 (0.32129)<br>HOMO–2 -> LUMO+2 (0.32129)<br>HOMO–3 -> LUMO+3 (0.12822)<br>HOMO–4 -> LUMO+4 (0.12822)
3.1057 eV	399.22 nm	1.9499	HOMO -> LUMO+1 (0.47204)<br>HOMO–1 -> LUMO+4 (-0.14530)<br>HOMO–1 -> LUMO (0.44016)<br>HOMO–2 -> LUMO+2 (0.14531)<br>HOMO–3 -> LUMO+2 (0.12343)<br>HOMO–4 -> LUMO+1 (-0.12343)
3.1057 eV	399.22 nm	1.9501	HOMO -> LUMO+2 (0.47204)<br>HOMO–1 -> LUMO+3 (0.14530)<br>HOMO–2 -> LUMO+4 (0.14530)<br>HOMO–2 -> LUMO (0.44016)<br>HOMO–3 -> LUMO+1 (0.12343)<br>HOMO–4 -> LUMO+2 (0.12343)
3.2595 eV	380.38 nm	0.0000	HOMO–3 -> LUMO (0.24847)<br>HOMO–2 -> LUMO+1 (0.38120)<br>HOMO–1 -> LUMO+2 (0.38120)<br>HOMO -> LUMO+3 (0.35211)
3.2595 eV	380.38 nm	0.0000	HOMO–4 -> LUMO (-0.24847)<br>HOMO–2 -> LUMO+2 (-0.38120)<br>HOMO–1 -> LUMO+1 (0.38120)<br>HOMO -> LUMO+4 (-0.35211)
3.3290 eV	372.44 nm	0.0000	HOMO–4 -> LUMO+1 (-0.14606)<br>HOMO–3 -> LUMO+2 (0.14606)<br>HOMO–2 -> LUMO+3 (-0.15531)<br>HOMO–1 -> LUMO (0.46885)<br>HOMO–1 -> LUMO+4 (0.15531)<br>HOMO -> LUMO+2 (-0.41360)

Table S6. Uncorrected and thermal-corrected (298K) energies of stationary points (Hartree).[a]

compound	E	E + ZPE	H	G
**[6]CN**	-2308.04746119	-2307.282516	-2307.239782	-2307.351706
**[7]CN**	-2692.74561642	-2691.852013	-2691.801972	-2691.931942
**[8]CN**	-3077.47382346	-3076.452172	-3076.394634	-3076.539354
**[9]CN**	-3462.16886695	-3461.019367	-3460.954276	-3461.117841
**D_{5d}-[10]CN**	-3846.88569576	-3845.607310	-3845.535005	-3845.712075
**[11]CN**	-4231.58031066	-4230.174649	-4230.094687	-4230.292301
**[12]CN**	-4616.28971037	-4614.755894	-4614.668476	-4614.880700
**[13]CN**	-5000.98410115	-4999.322873	-4999.227883	-4999.460027
**[14]CN**	-5385.68887218	-5383.899653	-5383.797228	-5384.044640
**[15]CN**	-5770.38363080	-5768.466931	-5768.356871	-5768.624163
**[16]CN**	-6155.08567522	-6153.041004	-6152.923481	-6153.205942
**C_s-[10]CN**	-3846.87497193	-3845.597614	-3845.525076	-3845.705930
**TS_{op}**	-3846.82850872	-3845.550923	-3845.479298	-3845.657558
**IM_{op}**	-3846.85136753	-3845.573522	-3845.501256	-3845.681643
**TS_{op}'**	-3846.83676970	-3845.559832	-3845.487840	-3845.669306
**TS_{pp}**	-3846.82633271	-3845.549815	-3845.477816	-3845.658656
**IM_{pp}**	-3846.86182086	-3845.583723	-3845.511471	-3845.691138
**TS_{pp}'**	-3846.83414227	-3845.557363	-3845.485410	-3845.665028

a) E: electronic energy; ZPE: zero-point energy; H (=E+ZPE+E_{vib}+E_{rot}+E_{trans}+RT): sum of electronic and themal enthalpies; G (=H–TS): sum of electronic and thermal free energies.

Table S7. Structural data of optimized structures of [8]-, [10]-, [12]-, and [16]CNs. Bond lengths (*a*, *b* (Å)), NICS(0) (ppm), the shielding constant of the hydrogen atom on 2-positions in extracted structure and CN (ppm), averaged bent angles (α, β (°)), and dihedral angles between the naphthalene ring and plane of CN (γ (°)). Because the shielding constant reproduced the observed upfield chemical shifts almost quantitatively, we conclude that the through-space magnetic shielding by the neighboring naphthalene rings caused the upfield chemical shifts of the protons at the 2,3-positions.

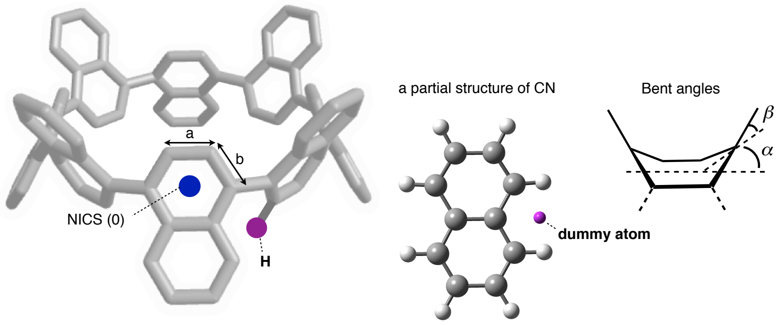

n in [*n*]CN	8	10	12	16
(2,3-position) (ppm)	6.93	7.04	7.18	7.33
(5,8-position) (ppm)	8.30	8.32	7.53	8.25
(6,7-position) (ppm)	7.48	7.51	8.30	7.54
a (Å)	1.406	1.406	1.406	1.407
b (Å)	1.388	1.387	1.386	1.385
NICS(0) (ppm)	–8.00	–8.08	–8.09	–8.11
Bent angle α (°)	11.6	8.4	7.1	5.5
Bent angle β (°)	12.5	10.2	8.5	6.4
Shielding constant (ppm)	–0.61	–0.51	–0.43	–0.31

Table S8. ^{1}H NMR chemical shift of ***C$_s$*-[10]CN** calculated by GIAO (ppm).

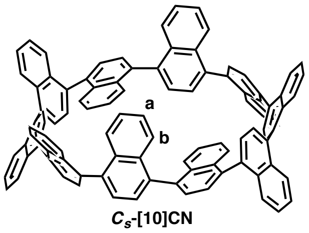

***C$_s$*-[10]CN**

	a	b
B3LYP/6-31G(d)//B3LYP/6-31G(d)	6.88	7.00
B3LYP/6-311+G(2d,p)//B3LYP/6-31G(d)	6.64	6.74

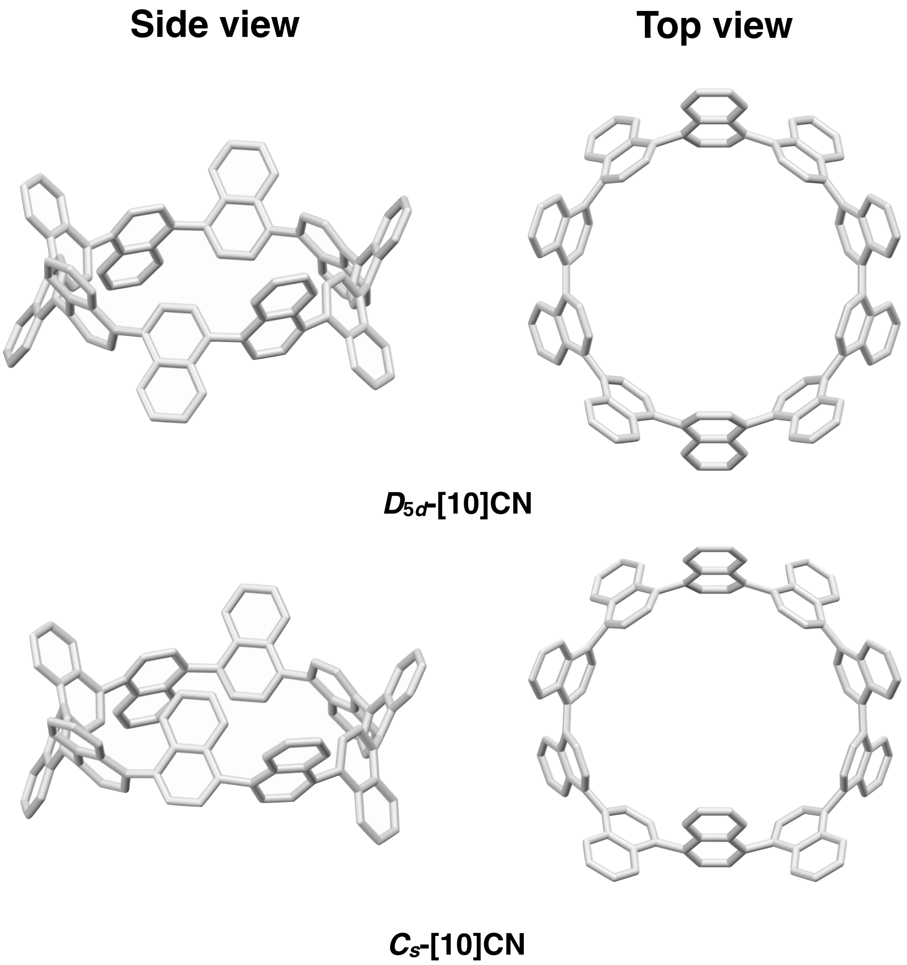

Figure S4. Optimized structures of ***D*$_{5d}$-[10]CN** and ***C*$_s$-[10]CN**.

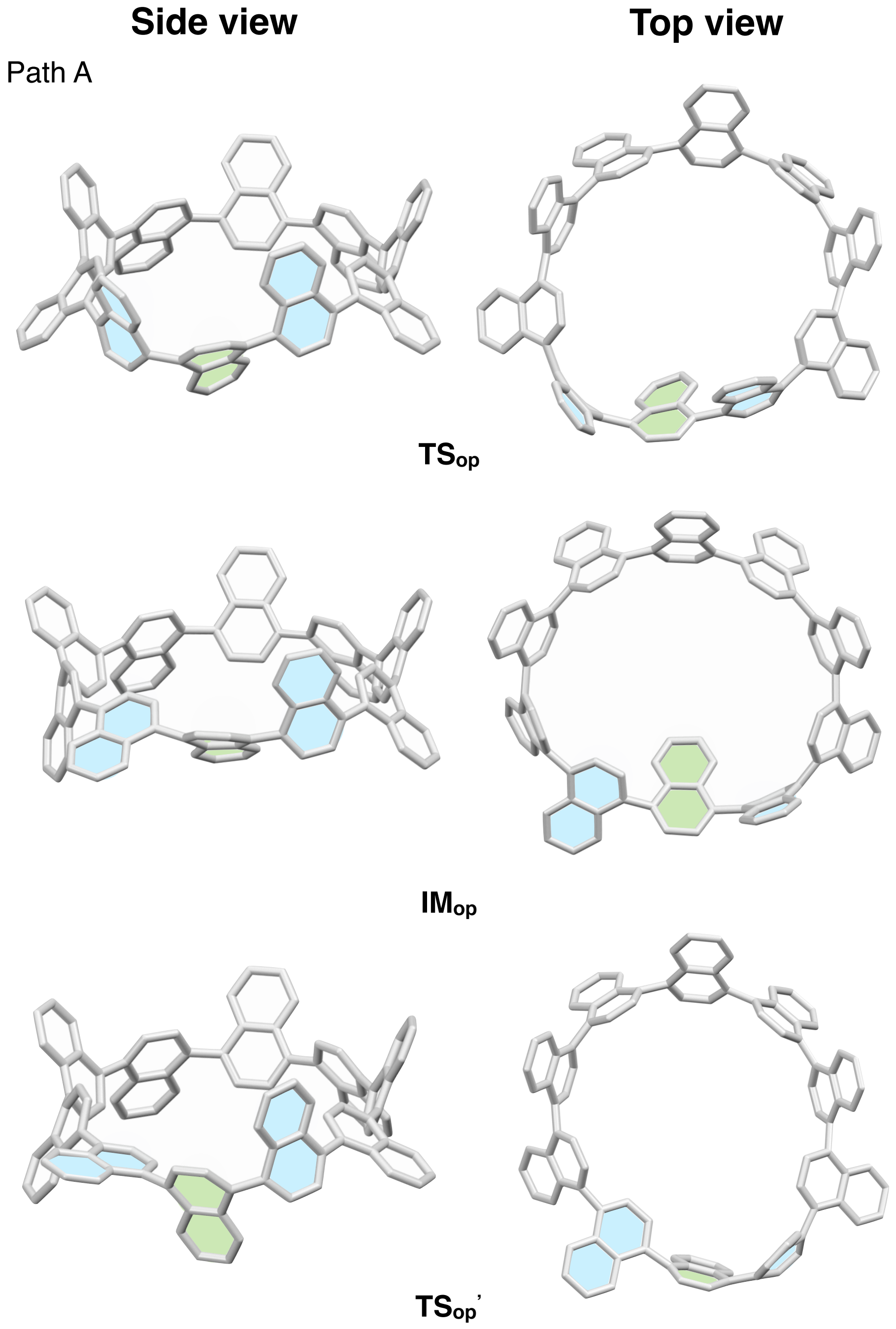

Figure S5. The transition states and the intermediate of the path A in the isomerization of [10]CN.

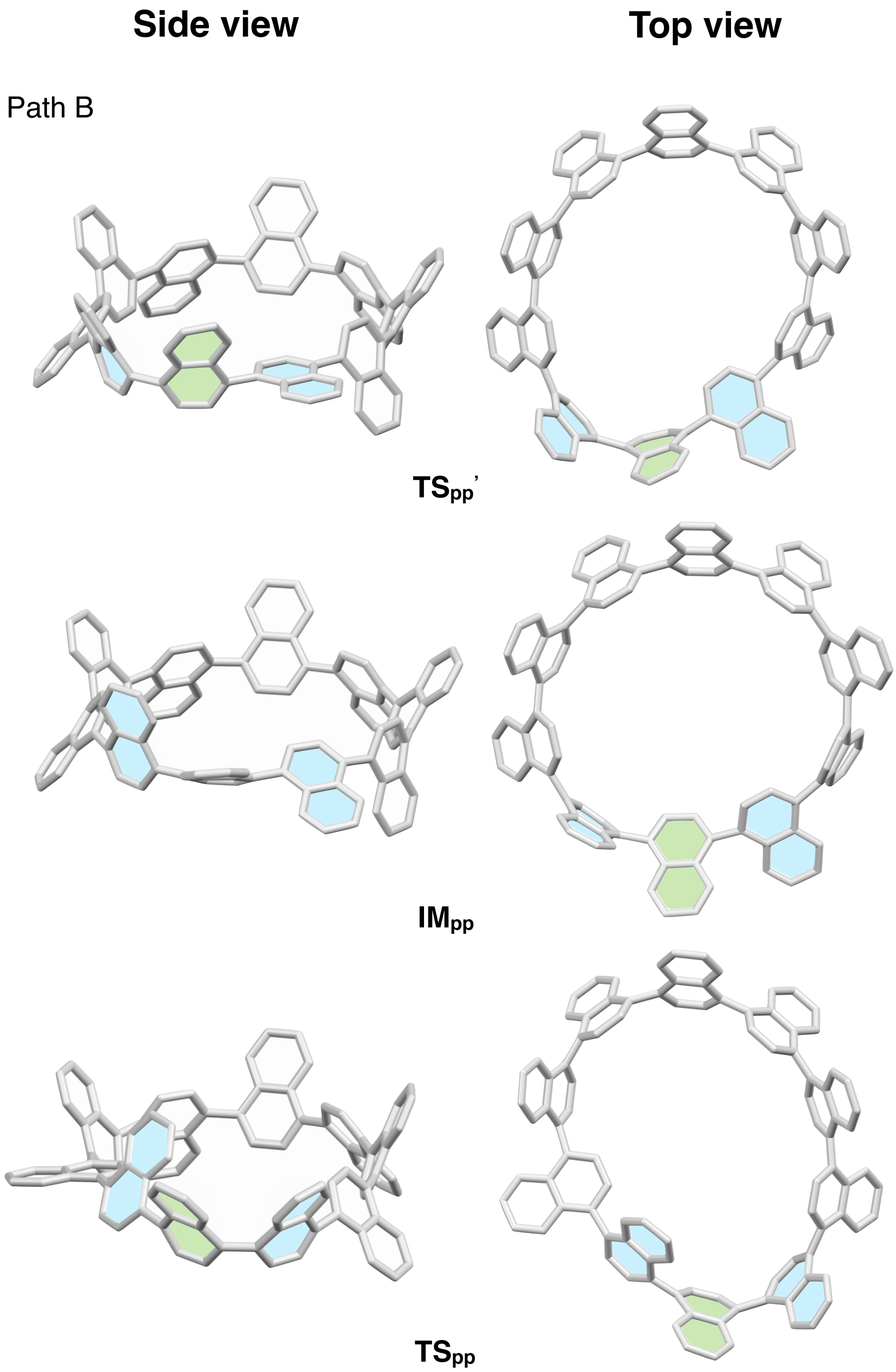

Figure S6. The transition states and the intermediate of the path B in the isomerization of [10]CN.

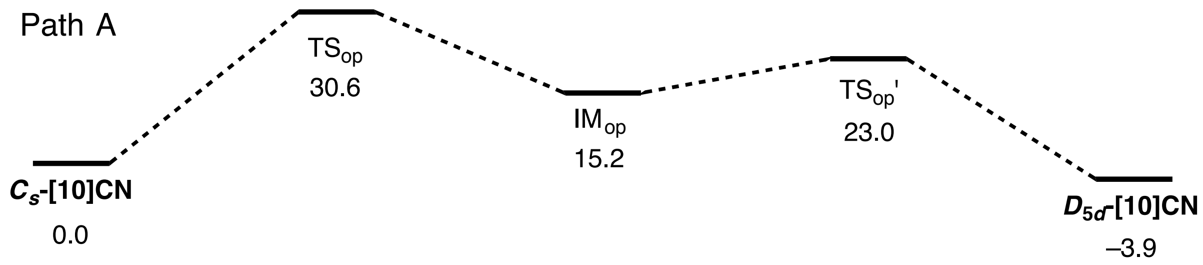

Figure S7. Isomerization pathway A and relative energies (Δ*G*/kcal·mol^{-1}) calculated by B3LYP/6-31G(d) level.

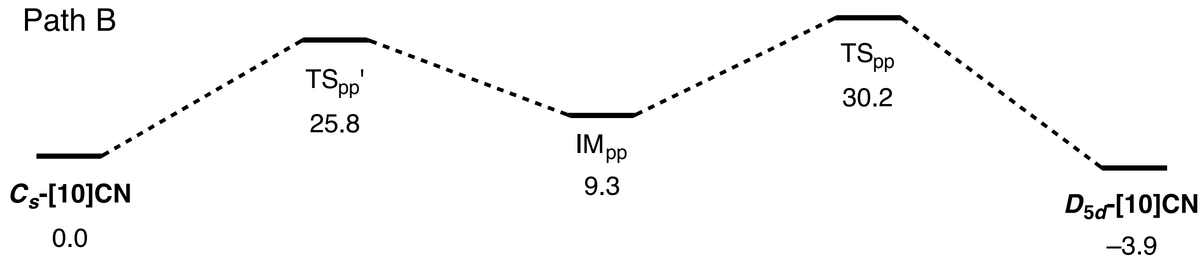

Figure S8. Isomerization pathway B and relative energies (Δ*G*/kcal·mol^{-1}) calculated by B3LYP/6-31G(d) level.

4. Kinetic study on the thermal isomerization from C_s-[10]CN to D_{5d}-[10]CN

Kinetics of the isomerization from $\boldsymbol{C_s}$**-[10]CN** to $\boldsymbol{D_{5d}}$**-[10]CN** was studied by monitoring the decreasing integration of $\boldsymbol{C_s}$**-[10]CN** in the ^{1}H NMR spectra in 1,1,2,2-tetrachloroethane-d_2 at 74.0, 77.8, and 81.0 °C (Figure S8). All reactions were conducted in NMR tube upon heating by oil bath under nitrogen. Reactions were stopped at each time for the ^{1}H NMR measurement by cooling the NMR tube in ice bath.

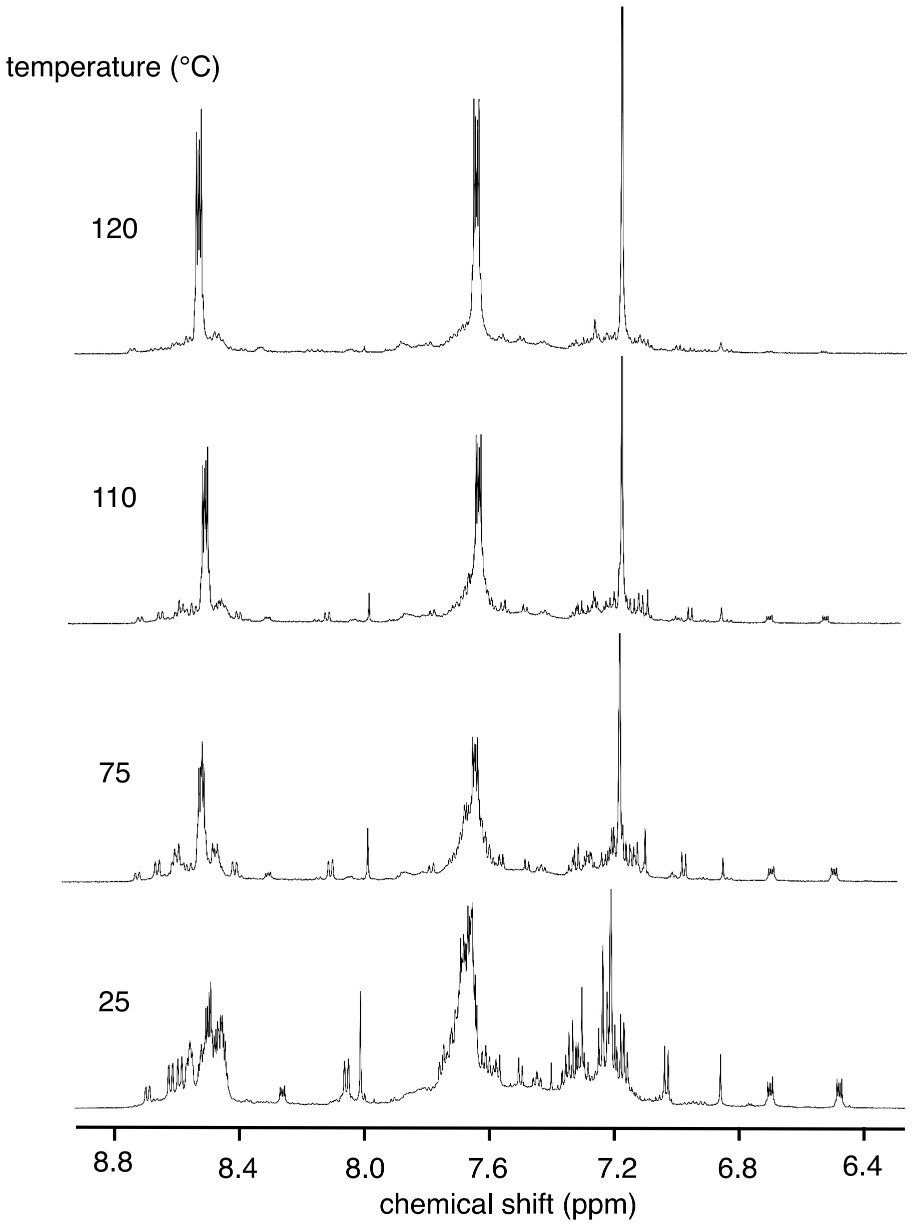

Figure S9. Conversion progress from $\boldsymbol{C_s}$**-[10]CN** to $\boldsymbol{D_{5d}}$**-[10]CN** at 25–120 °C monitored by ^{1}H NMR.

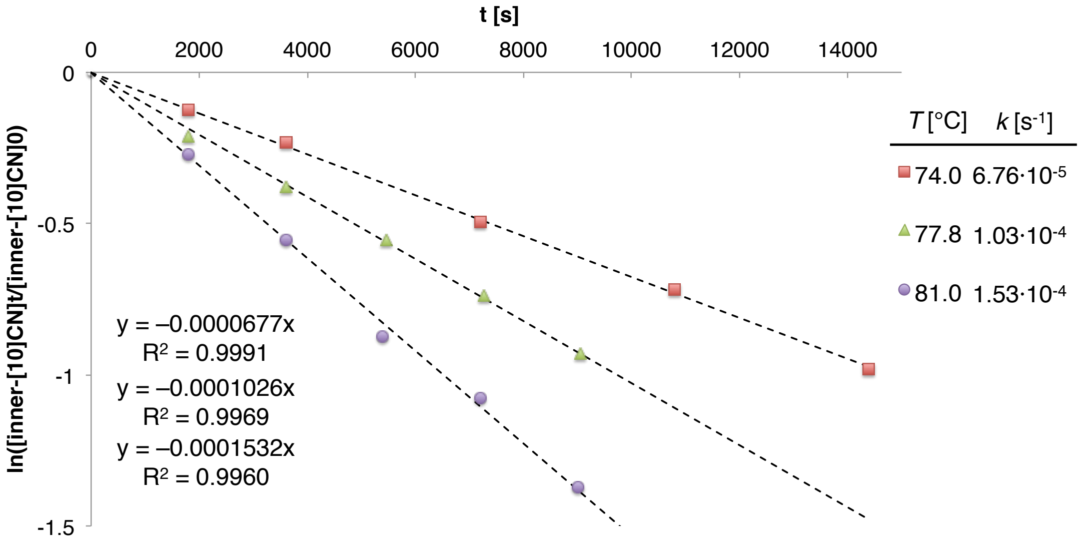

Figure S10. Plots of the decreasing integration of ***C_s*-[10]CN** in the ^{1}H NMR spectra in 1,1,2,2−tetrachloroethane−d_2 upon heating at 74.0, 77.8, and 81.0 °C.

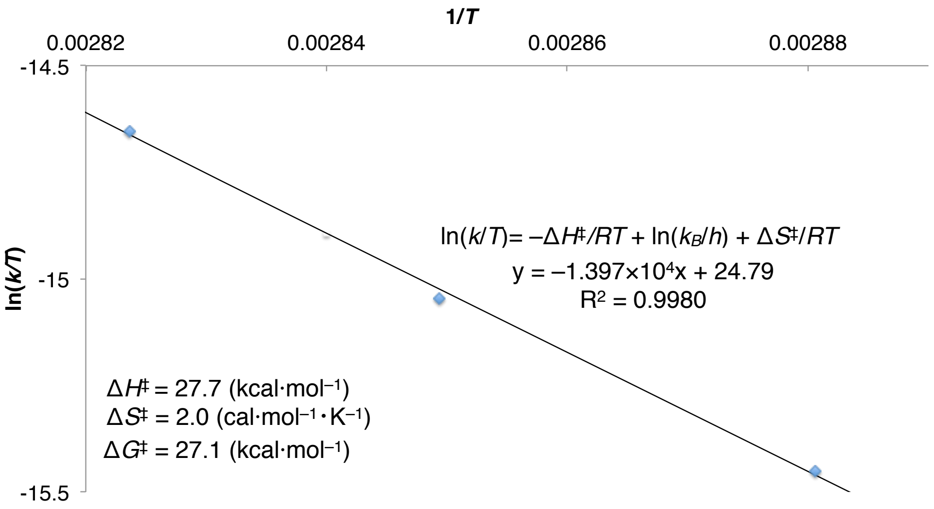

Figure S11. Eyring plot of the thermal isomerization from ***C_s*-[10]CN** to ***D_{5d}*-[10]CN**.

5. References

1) S. T. Horgen and P. A. Procopiou, Vinader Brugarolas, M. V. PCT Int. Appl. WO 2007071691 May 28, 2007.
2) A. Yagi, Y. Segawa and K. Itami, *J. Am. Chem. Soc.,* 2012*,* **134**, 2962.
3) M. J. Frisch, G. W. Trucks, H. B. Schlegel, G. E. Scuseria, M. A. Robb, J. R. Cheeseman, G. Scalmani, V. Barone, B. Mennucci, G. A. Petersson, H. Nakatsuji, M. Caricato, X. Li, H. P. Hratchian, A. F. Izmaylov, J. Bloino, G. Zheng, J. L. Sonnenberg, M. Hada, M. Ehara, K. Toyota, R. Fukuda, J. Hasegawa, M. Ishida, T. Nakajima, Y. Honda, O. Kitao, H. Nakai, T. Vreven, J. A. Montgomery, Jr., J. E. Peralta, F. Ogliaro, M. Bearpark, J. J. Heyd, E. Brothers, K. N. Kudin, V. N. Staroverov, R. Kobayashi, J. Normand, K. Raghavachari, A. Rendell, J. C. Burant, S. S. Iyengar, J. Tomasi, M. Cossi, N. Rega, J. M. Millam, M. Klene, J. E. Knox, J. B. Cross, V. Bakken, C. Adamo, J. Jaramillo, R. Gomperts, R. E. Stratmann, O. Yazyev, A. J. Austin, R. Cammi, C. Pomelli, J. W. Ochterski, R. L. Martin, K. Morokuma, V. G. Zakrzewski, G. A. Voth, P. Salvador, J. J. Dannenberg, S. Dapprich, A. D. Daniels, O. Farkas, J. B. Foresman, J. V. Ortiz, J. Cioslowski, and D. J. Fox, *Gaussian 09, Revision A.02*, Gaussian, Inc., Wallingford CT, 2009.
4) (a) A. D. Becke, *J. Chem. Phys*., 1993, **98**, 5648; (b) C. Lee, W. Yang and R. G. Parr, *Phys. Rev. B: Condens. Matter Mater. Phys*., 1988, **37**, 785.
5) (a) C. Gonzalez and H. B. Schlegel, *J. Chem. Phys.,* 1989, **90**, 2154; (b) C. Gonzalez and H. B. Schlegel, *J. Phys. Chem.,* 1990, **94**, 5523.
6) Y. Segawa, H. Omachi and K. Itami, *Org. Lett.,* 2010, **12**, 2262.

5. ^{1}H and ^{13}C NMR spectra of products

^{1}H NMR (600 MHz, $CDCl_3$) of **1b**

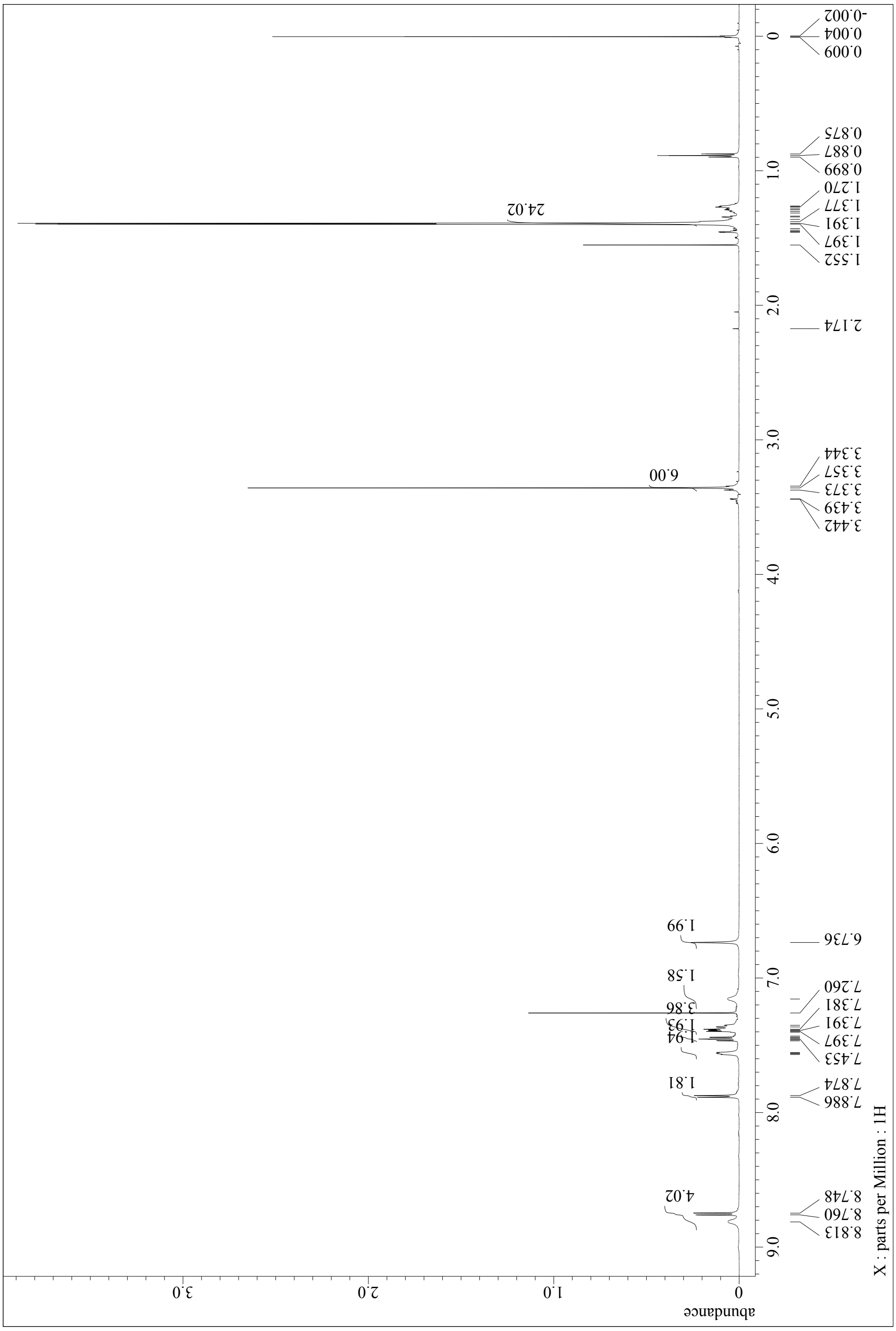

^{13}C NMR (600 MHz, CD_2Cl_2) of **1b**

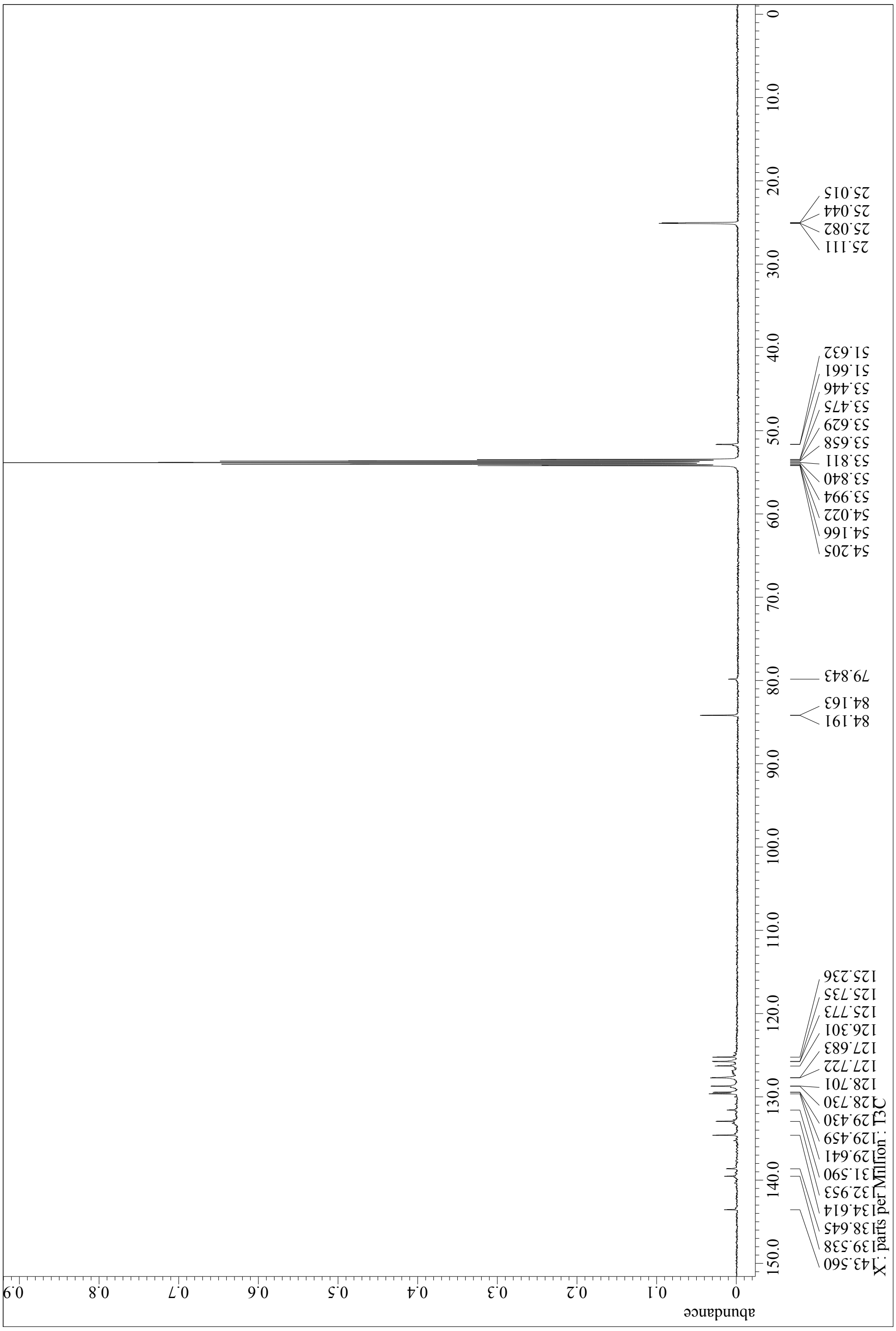

^{1}H NMR (600 MHz, CD_2Cl_2) of **2**

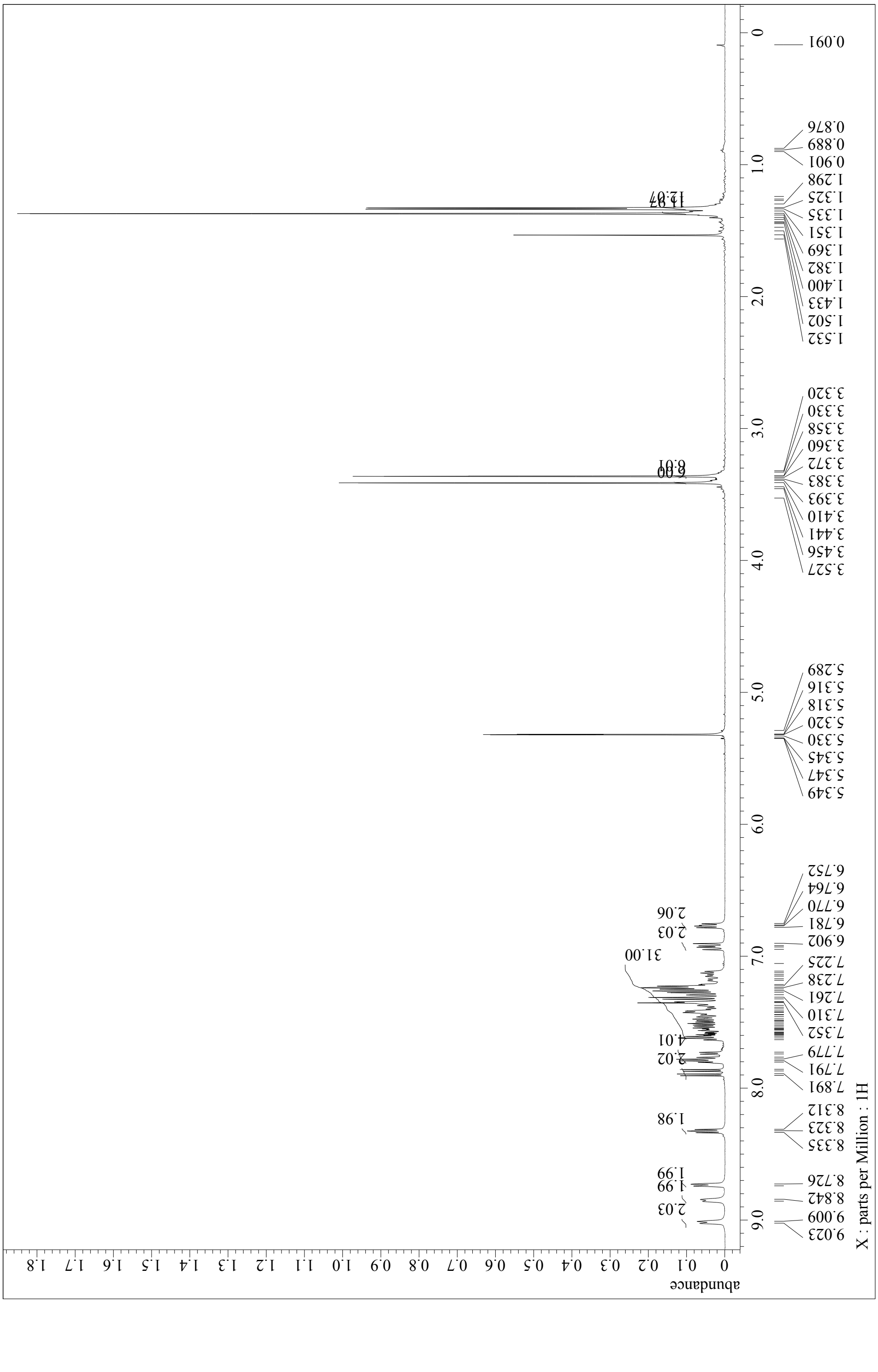

^{13}C NMR (600 MHz, CD_2Cl_2) of **2**

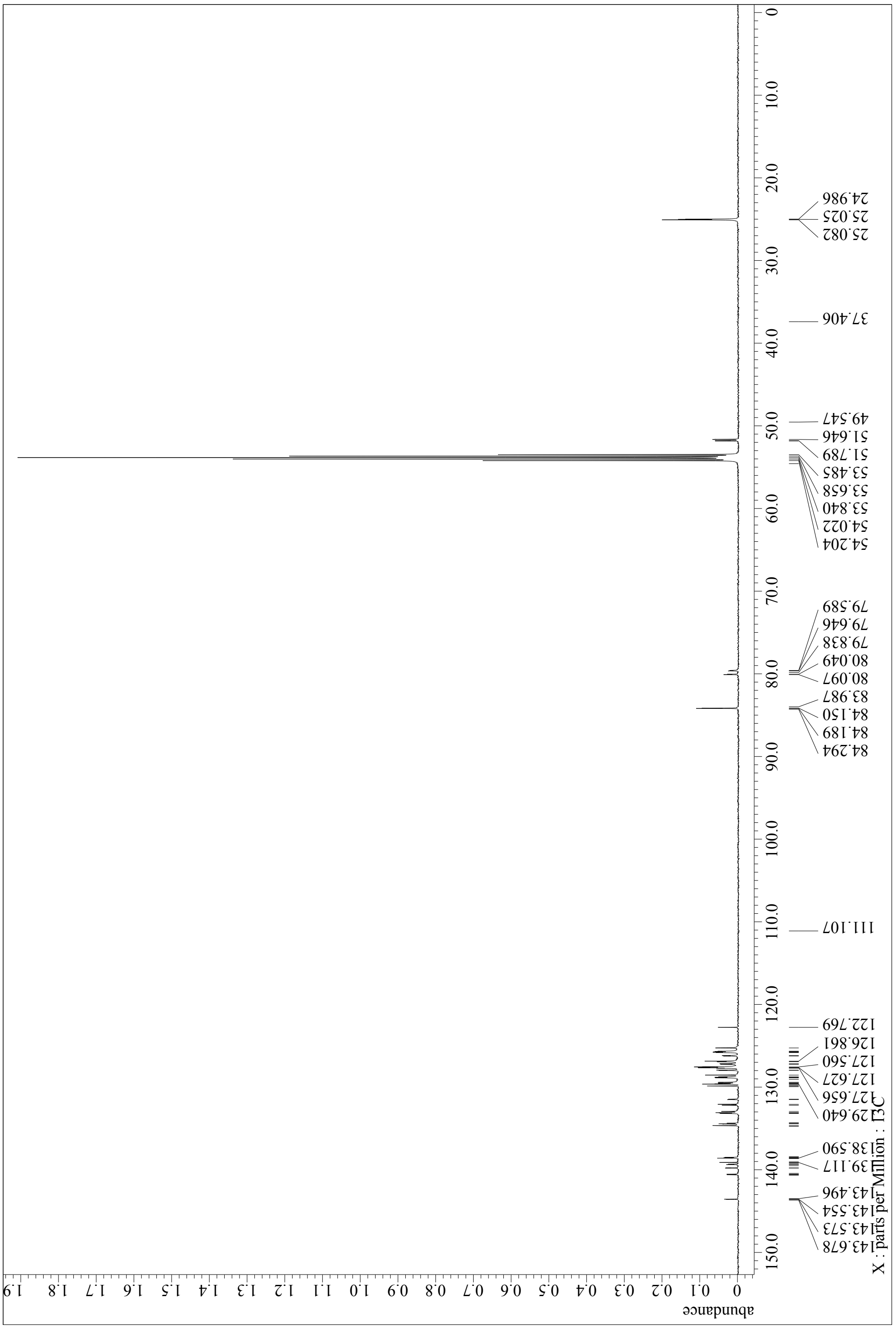

^{1}H NMR (600 MHz, CD_2Cl_2) of **3**

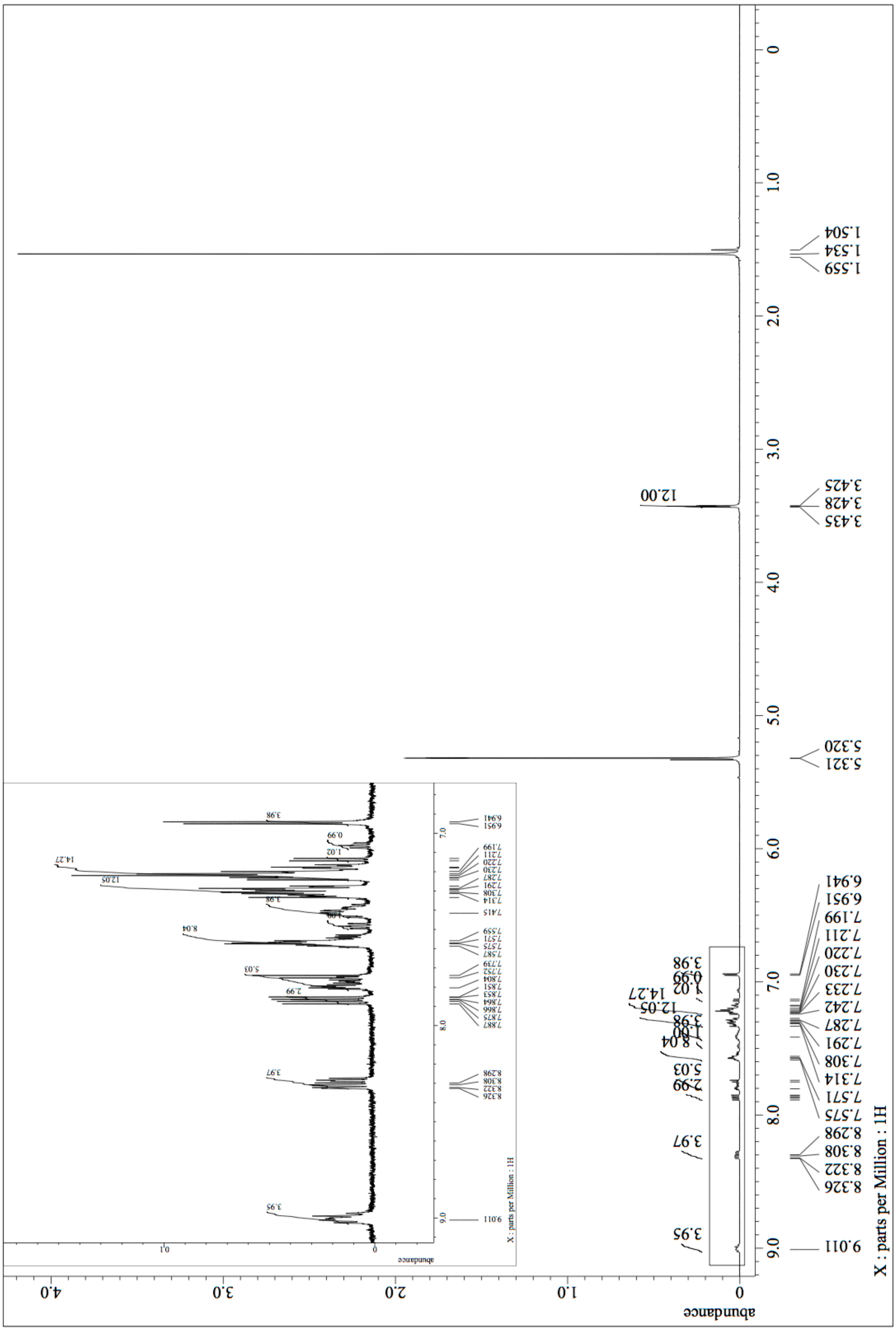

^{13}C NMR (600 MHz, CD_2Cl_2) of **3**

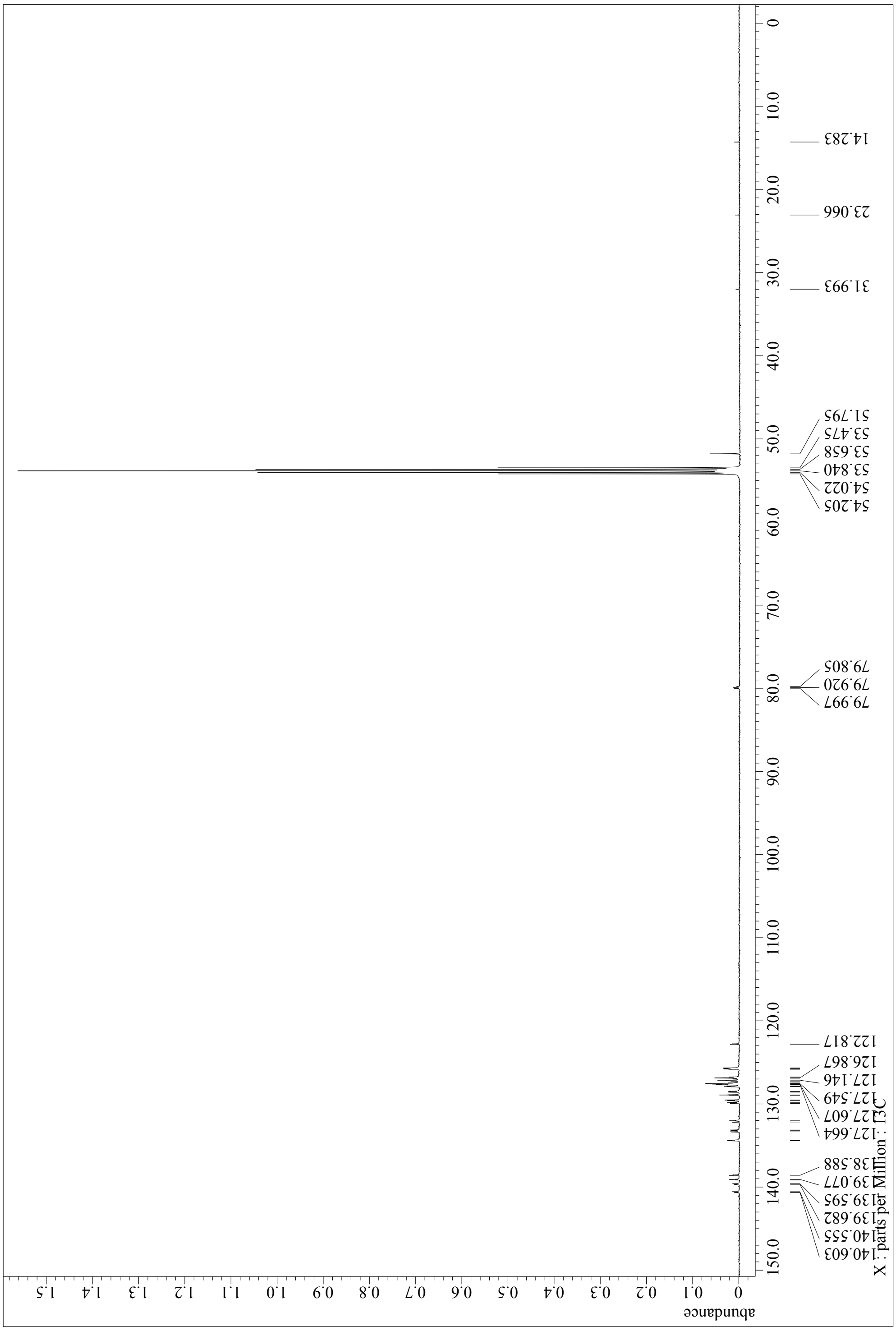

^{1}H NMR (600 MHz, CD_2Cl_2) of [8]CN

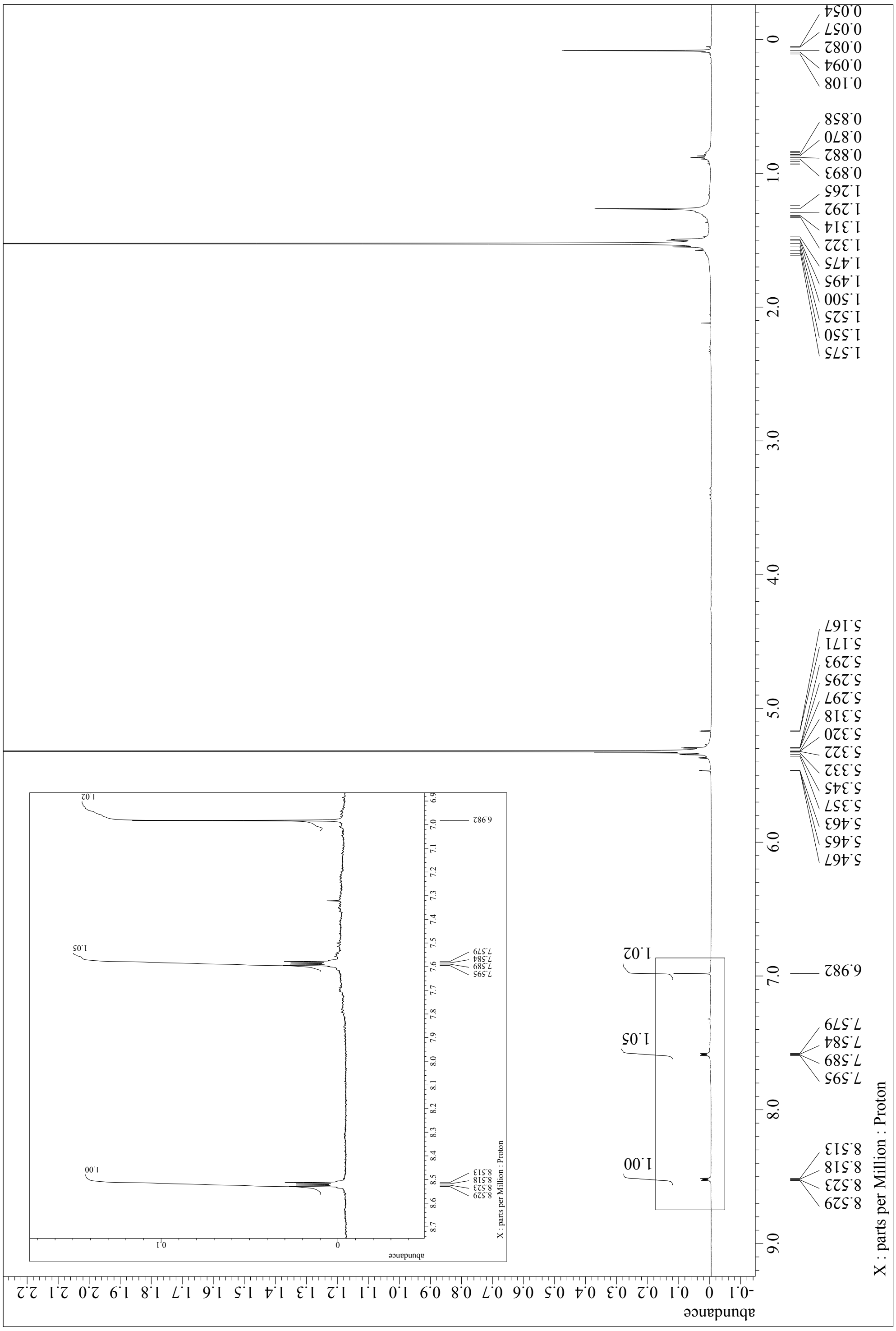

^{13}C NMR (600 MHz, CD_2Cl_2) of [8]CN

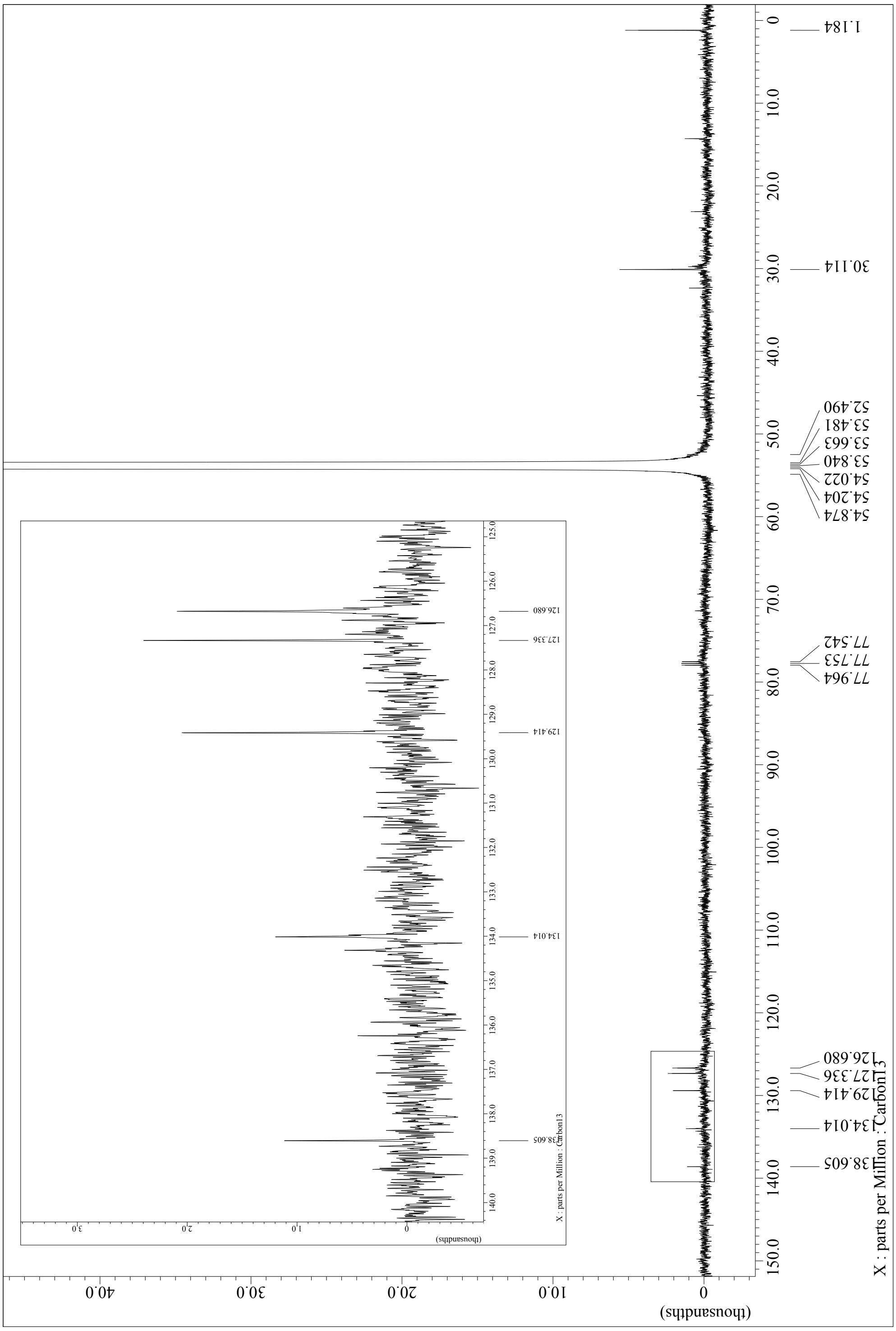

^{1}H NMR (600 MHz, CD_2Cl_2) of [12]CN

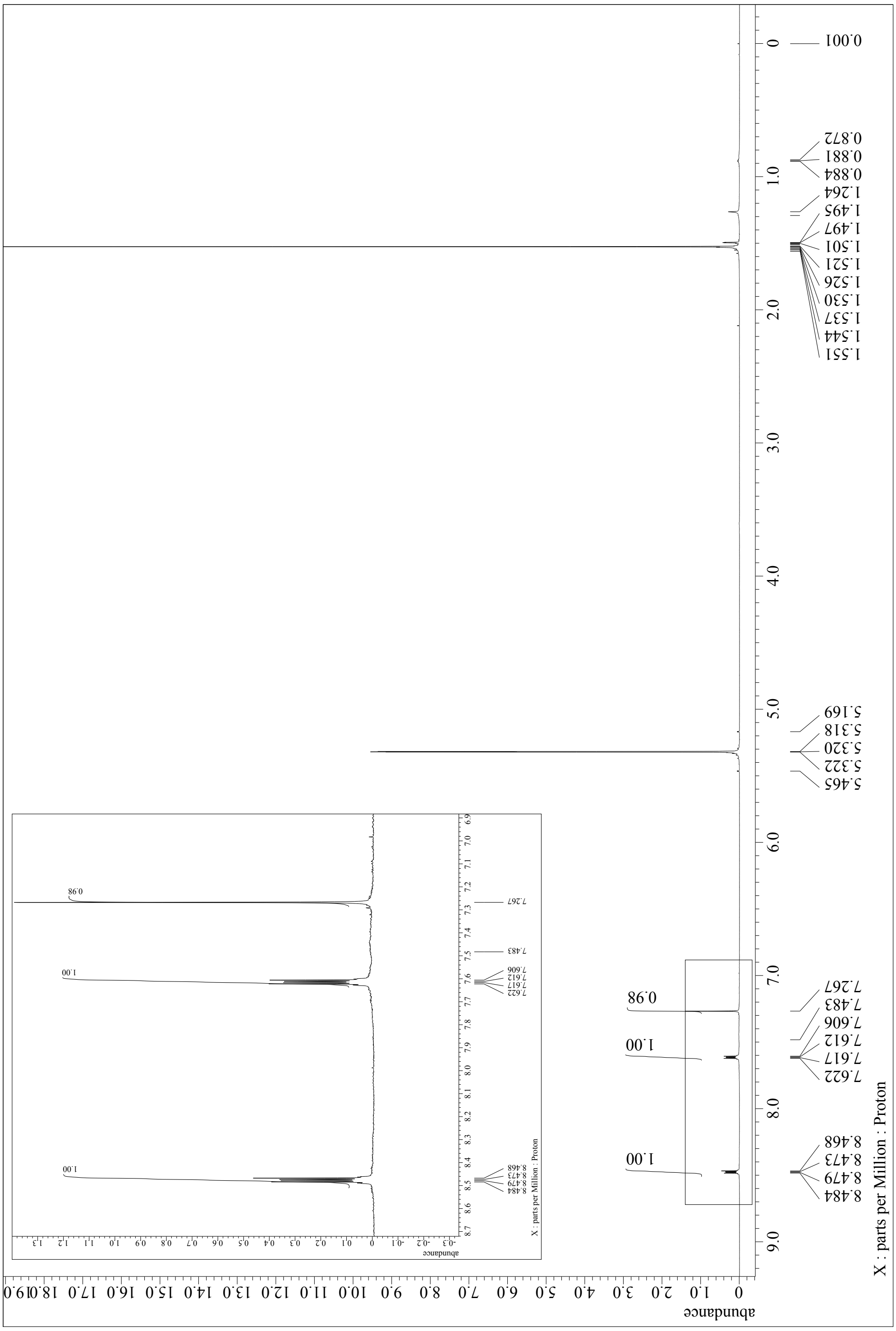

^{13}C NMR (600 MHz, CD_2Cl_2) of [12]CN

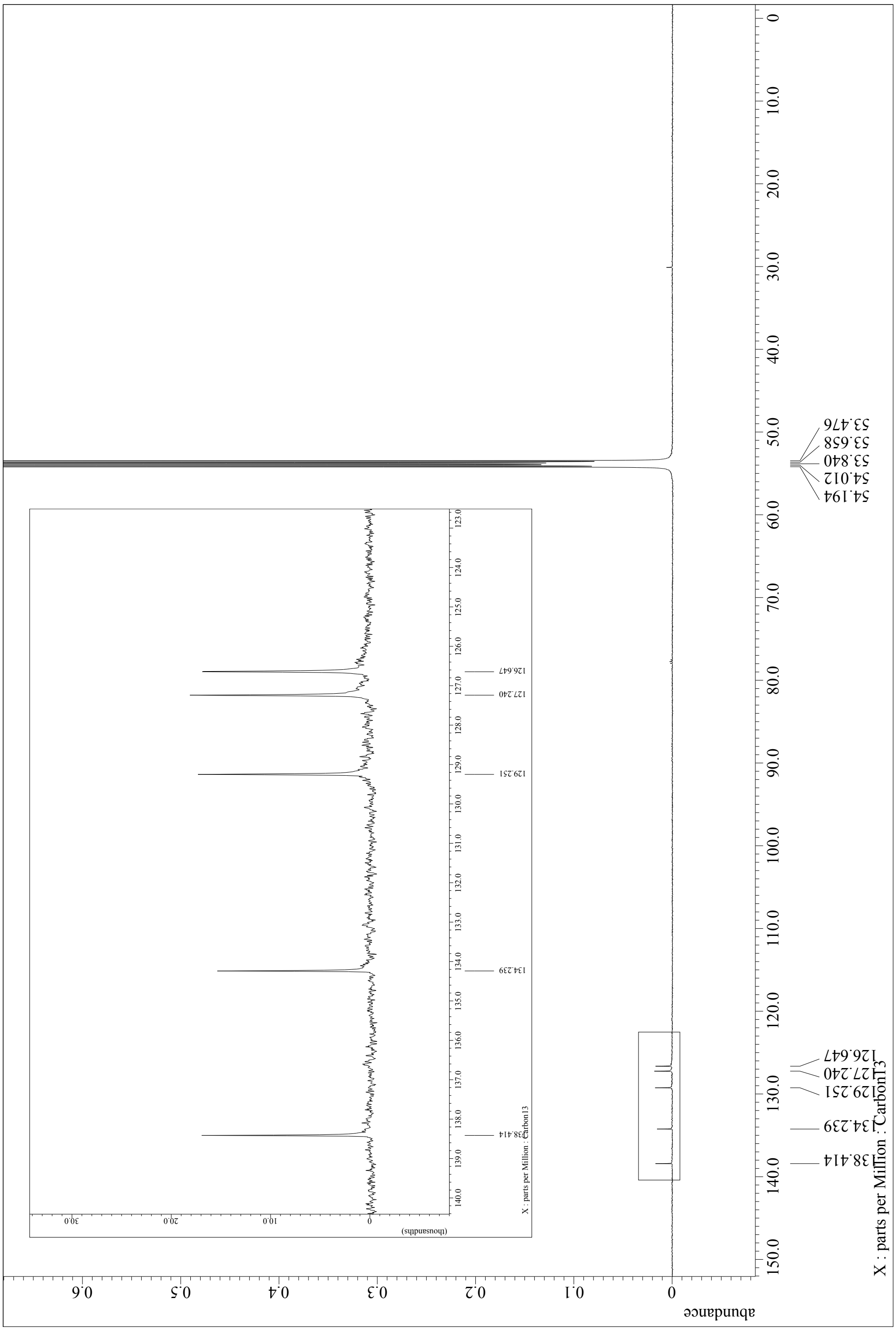

^{1}H NMR (600 MHz, CD_2Cl_2) of [16]CN

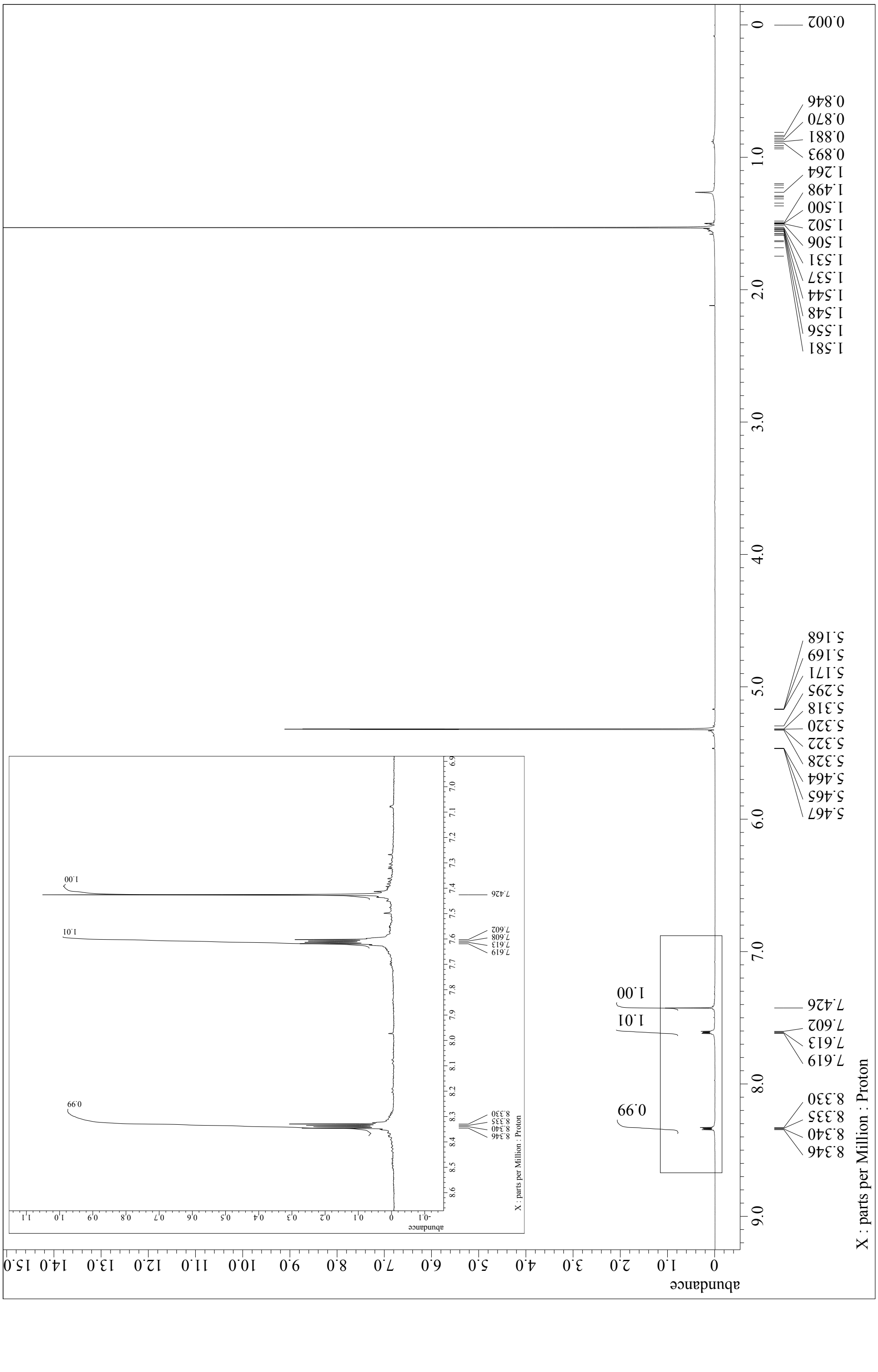

^{13}C NMR (600 MHz, CD_2Cl_2) of [16]CN

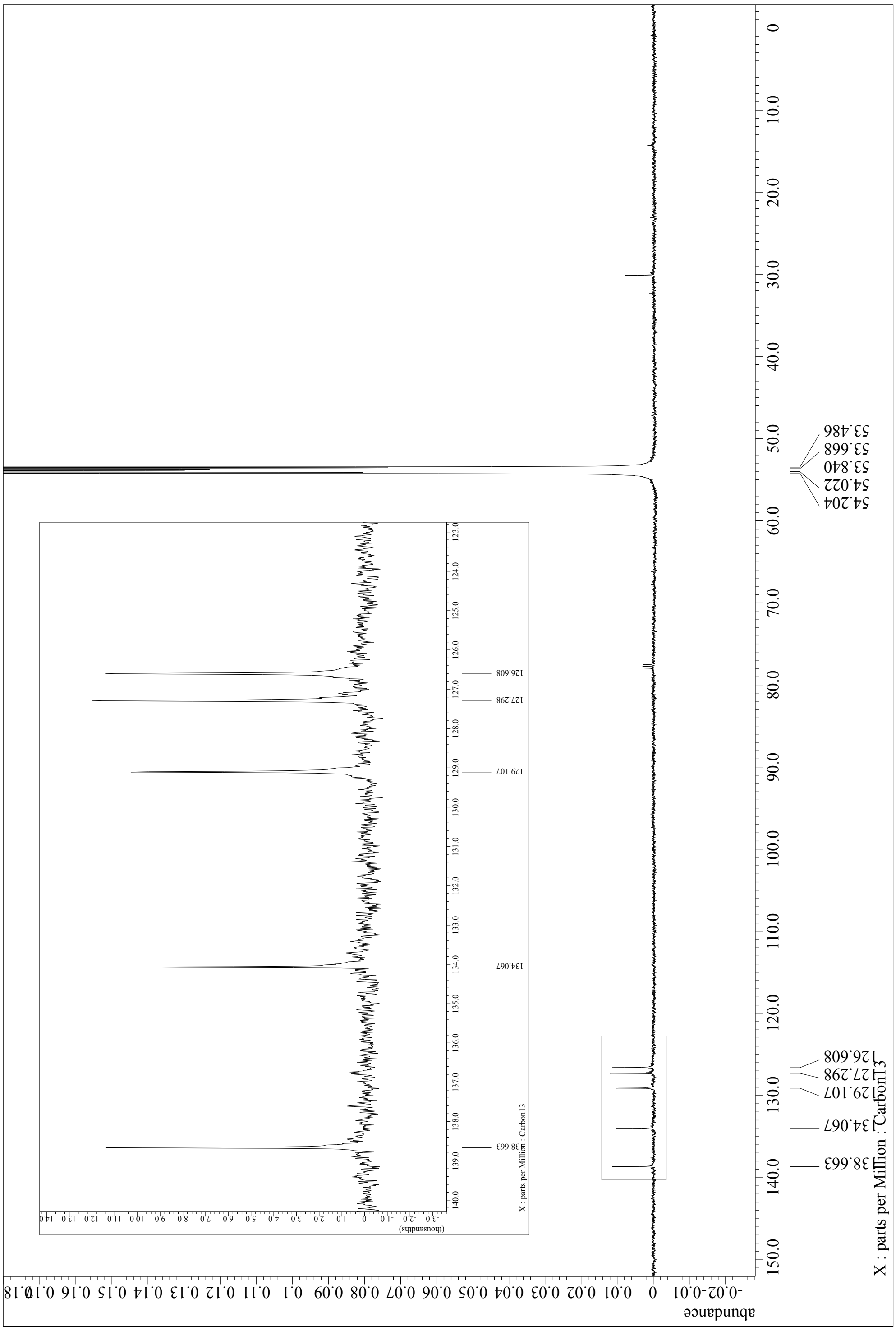

^{1}H NMR (600 MHz, CD_2Cl_2) of ***D*$_{5d}$-[10]CN**

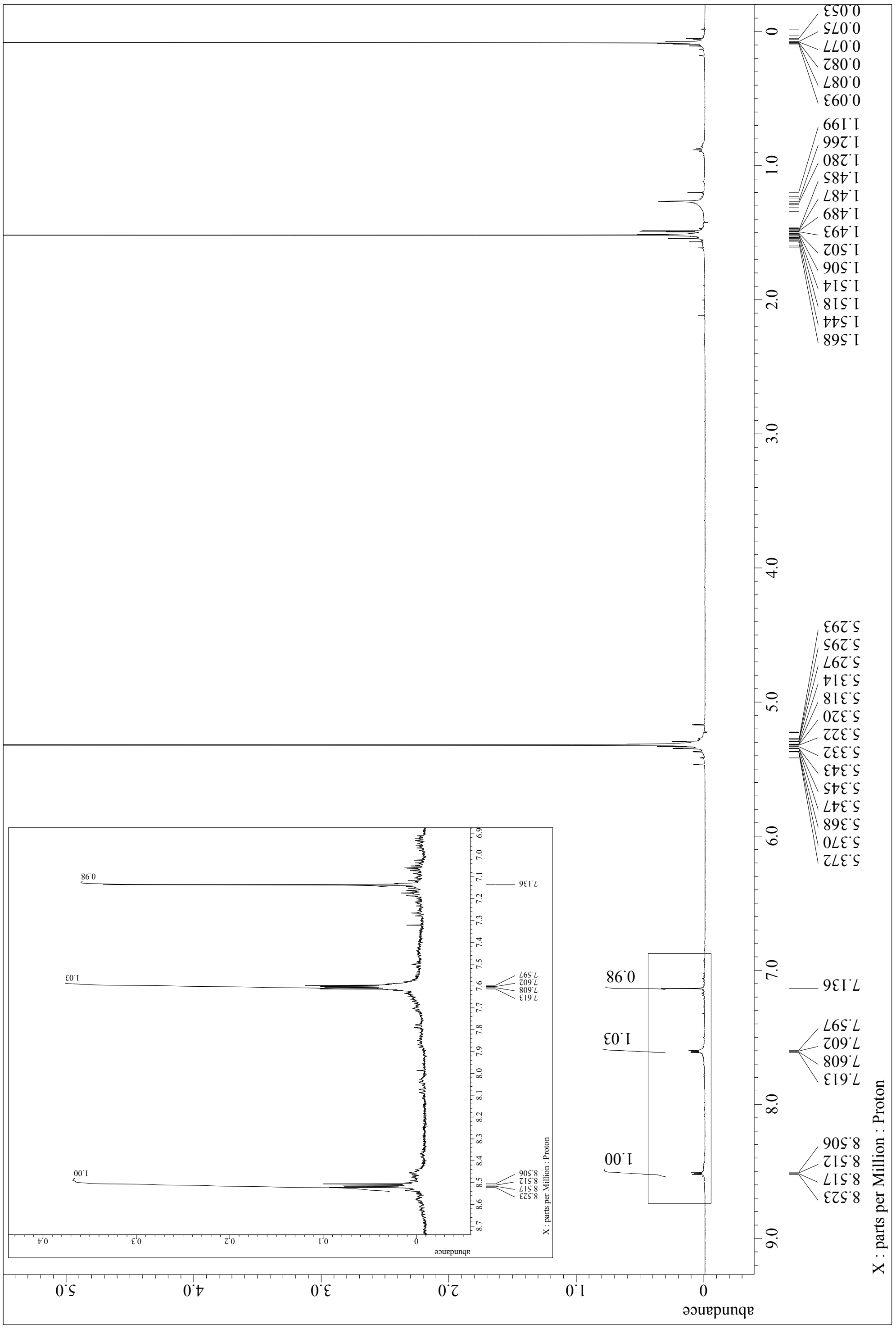

^{13}C NMR (600 MHz, CD_2Cl_2) of ***D$_{5d}$*-[10]CN**

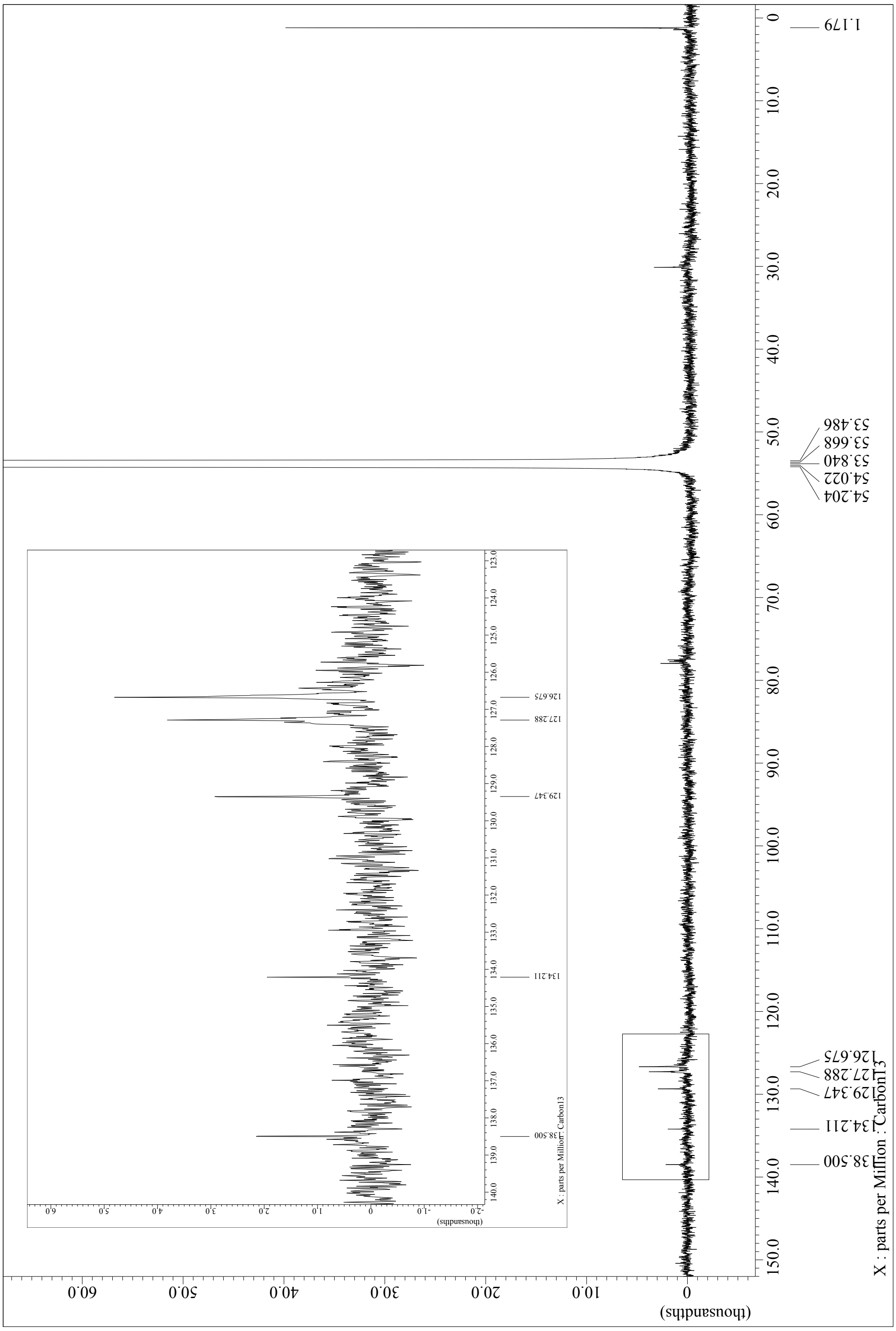

^{1}H NMR (600 MHz, CD_2Cl_2) of ***C**$_s$***-[10]CN**

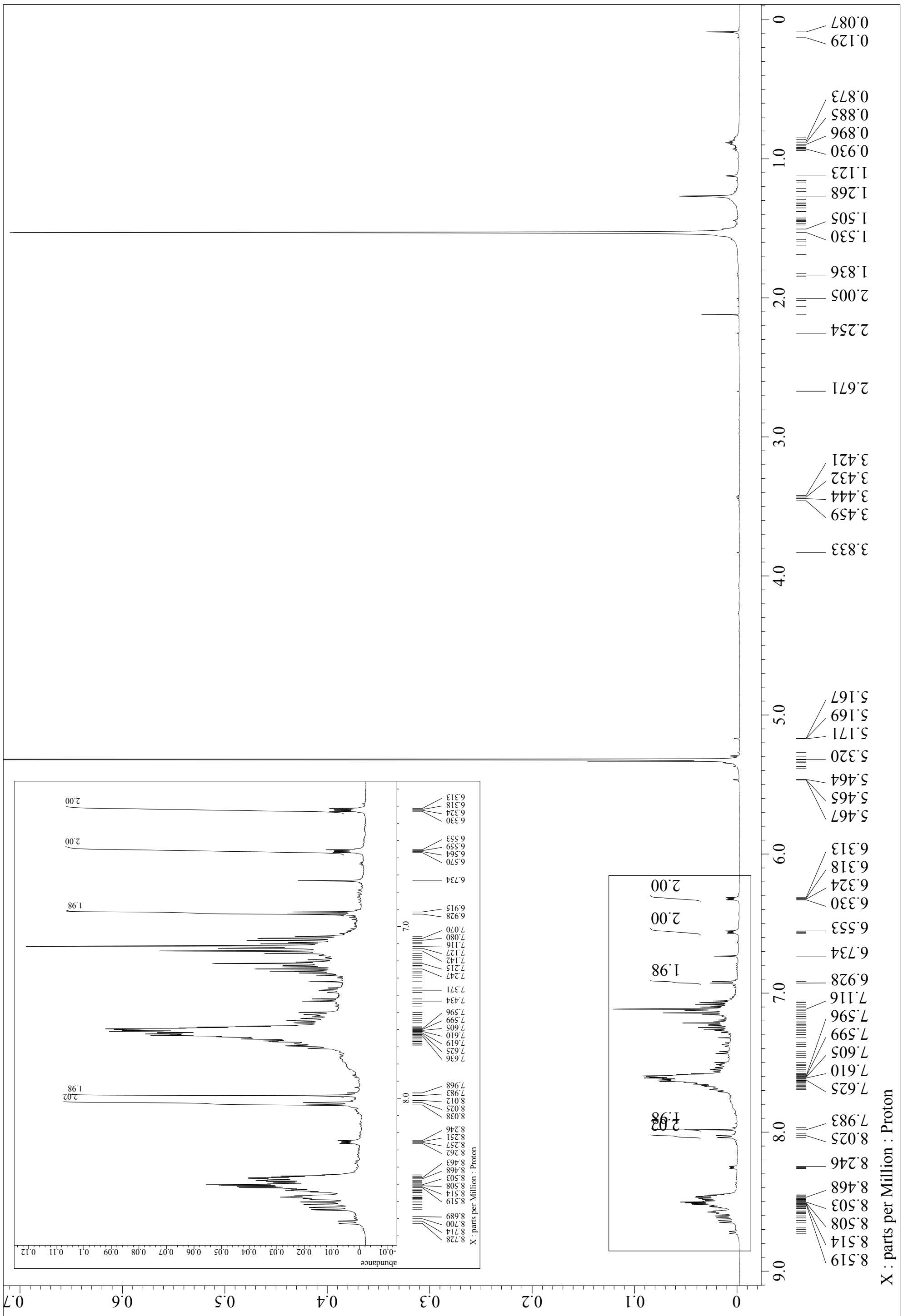

^{13}C NMR (600 MHz, CD_2Cl_2) of ***C$_s$*-[10]CN**

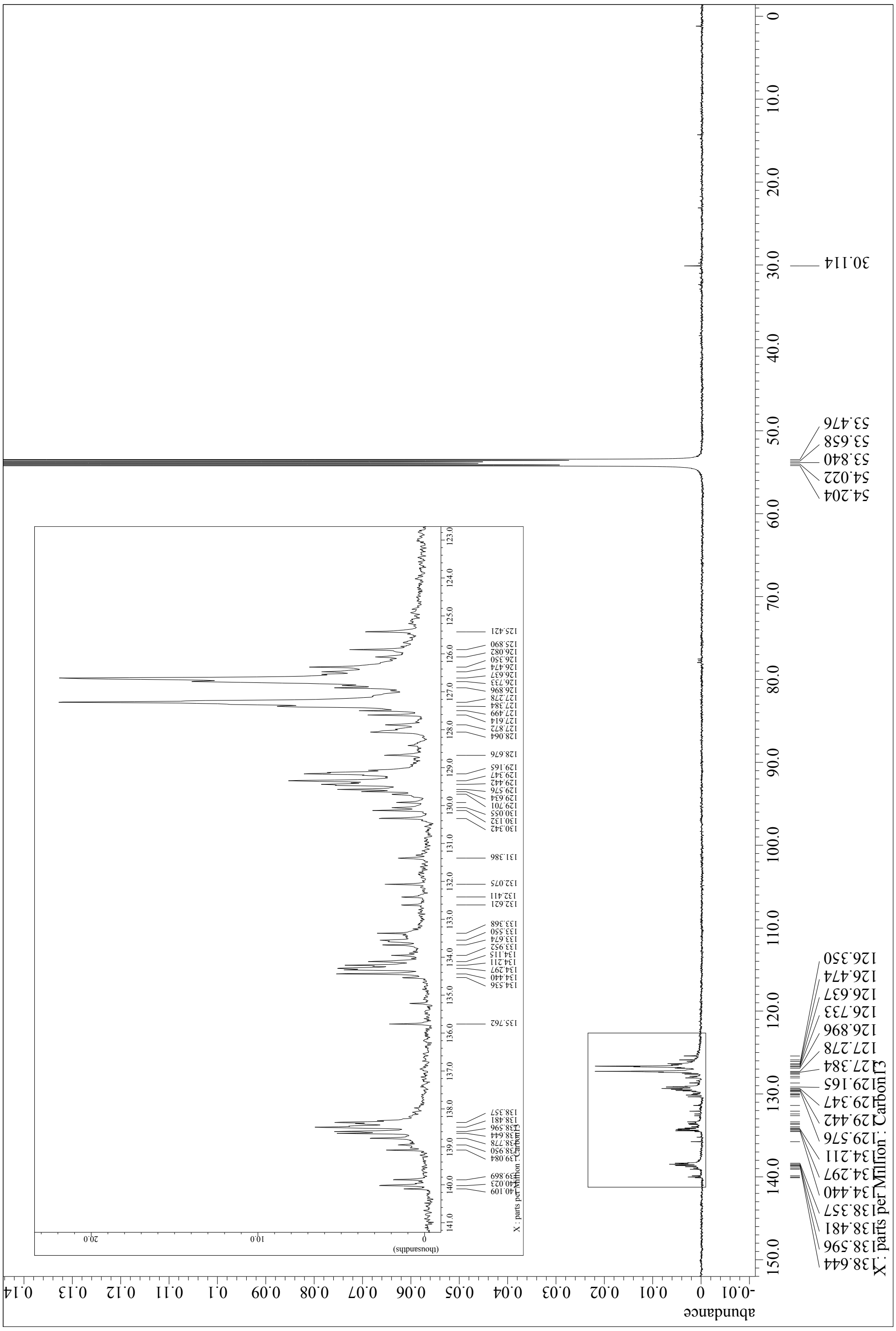

Supplement: Supplementary file 2 [file SC-008-C6SC04048A-s002.pdf]
